# PFKFB3 Mediated Glycolytic Reprogramming Drives Vascular Endothelial Injury Under Chronic Intermittent Hypoxia

**DOI:** 10.7150/ijbs.129280

**Published:** 2026-05-22

**Authors:** Shi Qi Li, Yi Wang, Liu Zhang, Ya Ru Yan, Fang Ying Lu, Xi Xi Chen, Ying Ni Lin, Jun Qi Lin, Jian Ping Zhou, Li Ming Lu, Qing Yun Li

**Affiliations:** 1 Department of Respiratory and Critical Care Medicine, Ruijin Hospital, Shanghai Jiao Tong University School of Medicine, Shanghai, 200025, China.; 2 Institute of Respiratory Diseases, Shanghai Jiao Tong University School of Medicine, Shanghai, 200025, China.; 3 Shanghai Institute of Immunology, Shanghai Jiao Tong University School of Medicine, Shanghai, 200025, China.

**Keywords:** obstructive sleep apnea, chronic intermittent hypoxia, endothelial inflammation, glycolysis, PFKFB3

## Abstract

Metabolic-inflammatory crosstalk is a hallmark of cardiovascular pathogenesis. Obstructive sleep apnea (OSA), characterized by chronic intermittent hypoxia (CIH), is an independent risk factor for cardiovascular diseases. While endothelial inflammation driven by CIH is pivotal in disease progression, the underlying metabolic mechanisms remain poorly defined. Our research shows that phosphofructo-2-kinase/fructose-2,6-biphosphatase 3 (PFKFB3), a key glycolytic activator, is markedly upregulated in endothelial cells (ECs) exposed to CIH, correlating with enhanced glycolysis, suppressed mitochondrial respiration, and amplified inflammatory responses. Endothelial-specific PFKFB3 deficiency or pharmacological suppression of PFKFB3 restores the glycolytic balance and alleviates vascular endothelial injury. Mechanistically, CIH enhances the expression of hypoxia-inducible factor 1α (HIF-1α), which regulates PFKFB3 expression. PFKFB3-induced production of lactate further promotes H3K18 lactylation (H3K18la), which in turn binds the PFKFB3 promoter, forming a positive-feedback loop. Disruption of the HIF-1α/PFKFB3 axis alleviates the inflammatory and glycolytic signatures of ECs. In conclusion, our findings identify PFKFB3 as a critical metabolic driver of endothelial inflammation under CIH, orchestrated through a HIF-1α-PFKFB3-H3K18la loop. These findings reveal novel pathogenic insights and potential therapeutic targets for OSA-associated cardiovascular diseases

## Introduction

Obstructive sleep apnea (OSA), characterized by repetitive partial or complete collapse of the upper airway during sleep, is a growing health burden worldwide^1^. Approximately 1 billion individuals are affected by OSA around the world^2^. OSA is a well-established risk factor for cardiovascular diseases (CVD), including hypertension, coronary artery disease, arrhythmia, heart failure, and stroke^3^. Chronic intermittent hypoxia (CIH), the principal intermediary mechanism underlying the deleterious consequence of OSA, has been posited to be related to oxidative stress, inflammation, sympathetic activation, hypercoagulability, and endothelial dysfunction, which in turn is responsible for OSA-related cardiovascular injury^4^.

Endothelial cells (ECs) constitute a crucial defensive barrier against CVD^5^. In the context of CVD, ECs become activated, leading to upregulated expression of adhesion molecules and secretion of cytokines and chemokines, thereby fostering a pro-inflammatory environment and facilitating leukocyte extravasation^6^. The mechanisms by which OSA/CIH mediates endothelial inflammation have been reported to comprise sympathetic activation-dependent catecholamine release^7^, internalization of CD59 and impaired endothelial complement inhibition^8^, ^9^, and activation of pro-inflammatory signaling pathway such as TLR4/NF-κB^10^, ^11^, HIF-1α/VEGF^12^, Erk1/2^13^, and TXNIP/NLRP3/IL-1β^14^. Nonetheless, the precise mechanisms of endothelial inflammation induced by CIH exposure in OSA remain incompletely understood.

Accumulating evidence highlights the importance of metabolic reprogramming in ECs activation and injury^15^. Notably, ECs exhibit a pronounced reliance on glycolysis, with nearly 85% of their ATP generated through the conversion of glucose to pyruvate in activated states^16^. Dysregulated glycolysis has been linked to endothelial dysfunction under various pathological stimuli, including lipoprotein(a), lysophosphatidylcholine, and transforming growth factor-β1^12^, ^17^, ^18^. Metabolomic studies have revealed disruptions in energy metabolism, particularly in anaerobic pathways, in patients with OSA and CIH-exposed rodent models^19^. However, the specific glycolytic reprogramming of ECs in response to CIH remains unexplored. Moreover, the key mediators connecting glycolytic rewiring to endothelial inflammation have yet to be elucidated.

6-phosphofructo-2-kinase/fructose2,6-bisphosphatase 3 (PFKFB3) serves as a central modulator of glycolytic flux. PFKFB3 modulates the synthesis of fructose 2,6-bisphosphate (F2,6P2), a potent allosteric activator of the rate-limiting glycolytic enzyme phosphofructokinase-1 (PFK-1), thereby sustaining glycolysis^20^. While much of the research surrounding PFKFB3 has centered on its roles in vessel sprouting and cancer development, its potential involvement in multiple biological processes remains largely unexplored^16^, ^20^. Emerging evidence indicates that PFKFB3-mediated glycolysis is instrumental in facilitating lipoprotein(a), lipopolysaccharide (LPS), or tumor necrosis factor-α (TNF-α) driven inflammation, highlighting its pivotal role in regulating a metabolic-inflammatory axis in ECs^21^. Drawing from these findings, we hypothesize that PFKFB3-driven endothelial glycolysis may play a key role in OSA-related vascular injury.

In the current study, we aim to characterize the glycolytic reprogramming of ECs in response to CIH and to explore the intricate interplay between metabolic dysregulation and inflammatory responses. Specifically, we seek to determine whether PFKFB3 acts as a critical mediator driving both inflammatory and glycolytic signatures of ECs. Finally, this project aims to explore the underlying mechanisms through which PFKFB3 directly regulates metabolic rewiring and inflammatory activation, thereby advancing our understanding of the pathogenesis of OSA-related cardiovascular complications.

## Materials and Methods

Detailed materials and methods were provided in the Supplementary Material.

### Study subjects

Patient samples were collected and analyzed in accordance with the Declaration of Helsinki and the study was approved by the ethics committee of Ruijin Hospital (Approval No.2019BC0001). All adult participants provided written informed consent to participate in this study. From July 2022 to March 2023, 110 participants with suspected OSA were randomly recruited from the sleep center of Ruijin Hospital. According to the Apnea Hypopnea Index (AHI), subjects were categorized into three groups, including the Non-OSA (AHI < 5), mild to moderate OSA (AHI 5-30), and severe OSA group (AHI ≥ 30). CVD risk was evaluated using the Framingham 2008 algorithm^22^. ELISA kits were used to measure concentration of PFKFB3(Aifang biological). Plasma lactate concentration was analyzed by a colorimetric L-Lactate assay kit (AAT Bioquest).

### Animal models of CIH and administration of AAV

All animal experimental and surgical procedures were approved by the Animal Care and Use Committee of Ruijin Hospital (Approval No. RJ2024072), in accordance with the guidelines for the care and use of laboratory animals implemented by the National Institutes of Health.

C57BL/6J mice and APOE^-/-^ mice on a C57BL/6 background (male, 8 weeks old) were exposed to cages via automated, computer-controlled gas exchange systems to achieve alternating 45s 6.5% O_2_ and 45s of 21% O_2_ cycles, 30 cycles/h for 8 h/day during daylight, while normoxic control (NC) mice were exposed to air (21% O_2_) in identical chambers.

For pharmacological treatment, mice were administered 3PO (20mg/kg; i.p., daily), SAC (5 mg/kg; i.p., daily), or 2-ME (5 mg/kg; i.p., every two days) 1 hour before CIH challenge.

For *in vivo* vascular endothelial-specific PFKFB3 over-expression or knockdown, custom-made adeno-associated viral vector (HBAAV2/VEC) carrying mouse Pfkfb3 or mir30 targeting mouse Pfkfb3 with a Tie promoter and its negative control (AAV-control) were obtained from Hanbio^18^. A single injection of AAV- Pfkfb3 (High), AAV- Pfkfb3 (Low), or AAV-control at a dose of 1 × 10^11^ viral genomes was administrated into mice via intravenous injection. Bioluminescence imaging was performed at 4 weeks after AAV injection, following the instructions of the IVIS Lumina XR small animal optical imaging system (PerkinElmer). Primary aortic ECs and non-ECs were isolated using CD31 magnetic beads (Miltenyi Biotec), and Pfkfb3 expression was assessed by Western blot.

### Blood pressure, immunofluorescence and Oil-Red-O staining

Blood pressure was consecutively measured three times in steady-state conditions using the tail-cuff method (Shanghai Meilisai Life Science Co., Ltd). At the end of the experimental periods, mice were anesthetized with inhaled 4% isoflurane and euthanized for tissue collection. Hearts and 1/2 of the ascending aortas were fixed in 4% paraformaldehyde and processed for OCT embedding, from which slices of aortic root were stained with Oil Red O. Immunofluorescent staining was conducted in our previous reports^14^. Briefly, aortic sections were embedded in paraformaldehyde and cut into 10 µm slices. The aortas were stained for PFKFB3, IL-6, ICAM-1, CD31, and nuclei/DAPI. Optical samples were observed under a Leica TCS SP8 confocal microscope (Leica Microsystems, Wetzlar, Germany) and fluorescent intensity was quantified using ImageJ.

### Single cell suspensions from mouse aorta and flow cytometry

Abdominal aortas were digested and resuspended for flow cytometry^23^. Single cell suspensions were stained with a cocktail of antibodies (10^6^ cells-100ul cocktail) against live-dead, CD45, CD11b, CD11c, Ly6C, Ly6G, F4/80, B220, CD3, CD4, CD8, CD86 (all from BD Pharmingen). Cells were analyzed by BD LSRFortessa X20 and FlowJo Software (Tree Star Inc.).

### Cell models of CIH and plasma stimulation

Primary human umbilical vein endothelial cells (HUVECs) were exposed to cages where O_2_ concentration oscillated between 1% O_2_ for 5 min and 21% O_2_ for 5 min via the automated, computer-controlled gas exchange system for 24 h^14^.

For the plasma stimulation model, HUVECs were incubated with 20% plasma from Non-OSA (N = 10), mild to moderate OSA (N = 10), and severe OSA (N = 10), which were age, sex and body mass index matched. For lactate-only treatment, HUVECs were treated with lactate at the average concentrations of non-OSA, mild-moderate OSA, or severe OSA plasma.

In experiments including inhibitors, 2 µg/mL 3PO (MCE, HY-19824), 10µM SAC (MCE, HY-N0319), 1 uM 2-ME (MCE, HY-12033), 10 mM Oxamate (MCE, HY-W013032A), or 10 mM 2-deoxyglucose (2DG, Agilent) was added 1 hour prior to CIH exposure.

For transductions, HUVECs were transduced with either pLV-PFKFB3 for over-expression, pLV-shPFKFB3 to silence PFKFB3 expression, or pLV-control followed by antibiotic selection. For RNAi experiments, HUVECs were transfected with HIF-1α siRNA or negative control siRNA (Aisen) using Lipofectamine 3000 (Invitrogen) according to the manufacturer's instructions.

### RNA sequencing, RT-qPCR and western blot analysis

Total RNA was extracted and sequenced used DNBSEQ platform. Differentially expressed genes (DEGs) with a false discovery corrected p < 0.05 were used for further analysis on the platform of Dr.Tom (BGI). Heatmaps were made by TBtools. For RT-qPCR, primer sequences were outlined in Supplementary Table.2. For western blot analysis, primary antibodies were outlined in Supplementary Table 3.

### Monocyte-endothelial adhesion assay

HUVECs were exposed to NC or CIH in the presence or absence of inhibitors. 4×10^5^ THP-1 cells per well were added into plates and incubated at 37 °C in 5% CO_2_ for 1 h, followed by washing to remove non-adhered monocytes. The number of adhered monocytes was counted using Zeiss Axio Vert.A1 microscope (Zeiss, Germany).

### Seahorse flux analysis

The Seahorse XFe96 analyzer (Agilent Technologies) was used for bioenergetic analysis. Extracellular acidification rates (ECAR) were measured by Seahorse XF Glycolysis Stress Test Kit. OXPHOS was determined by Seahorse XF Cell Mito Stress Test Kit. For the plasma stimulation model, HUVECs were incubated in endothelial cell medium with 20% plasma from different group and analyzed using Seahorse XF Glycolytic Rate Assay Kit. The Seahorse XFe96 analyzer was operated using the WAVE Software.

### CUT&Tag-Seq and CUT&Tag-qPCR

CUT&Tag was performed by Hyperactive Universal CUT&Tag Assay Kit for Illumina Pro (TD904, Vazyme) according to the manufacturer's instructions. The library was quantified and sequenced on an Illumina HiSeq/Novaseq instrument (Illumina, San Diego, CA, USA). For analysis of the CUT&Tag data, clean reads were aligned to the human reference genome (GRCh38.109) via software Bowtie2 (version 2.2.6). Peaks quality control, peaks calling and peaks annotation were analyzed by MACS (V2). For data visualization, the Integrative Genomics Viewer (IGV) was utilized. The primer sequences for CUT&Tag-qPCR were listed in Supplementary Table.2.

### Luciferase reporter assay

To assess the functional role of H3K18la in PFKFB3 transcription, a dual-luciferase reporter assay was performed. The wild-type (WT) PFKFB3 promoter sequence and a mutant (MUT) version lacking putative H3K18la-responsive elements were cloned into the pGL4-Basic luciferase reporter vector (Promega). HUVECs were co-transfected with WT or MUT reporter constructs and a Renilla luciferase control vector (pRL-TK, Promega) using Lipofectamine 3000 (Invitrogen). After 48 h, cells were exposed to NC or CIH for an additional 24 h, with or without Oxamate or 2DG treatment. Luciferase activity was measured using the Dual-Luciferase Reporter Assay System (Invitrogen) and normalized to Renilla activity.

### Bioinformatics analysis

Correlation analysis of gene expression of aortas was based on the Genotype-Tissue Expression (GTEx) database using the online tool GEPIA (Gene Expression Profiling Interactive Analysis).

Single-cell RNA sequencing (scRNA-seq) analysis was based on the GSE159677 dataset^24^. The data was analyzed by the R package 'Seurat' (v4.0.2)^25^, which was used to perform filtering, normalization, dimensionality reduction, clustering, and differential expression analysis. Cell clusters were annotated by using both the R package 'SingleR' (v1.6.1)^26^. Next, ECs with above-average PFKFB3 expression were defined as PFKFB3-high ECs, and the rest as PFKFB3-low ECs. Differentially expressed genes were identified with adjusted *P* values less than 0.05.

### Statistical analysis

Statistical analyses were performed using GraphPad Prism (v.8.0, GraphPad Software, La Jolla, CA, USA) and SPSS software (v.22.0, SSPS Inc, Chicago, IL). Normal distribution was assessed using the Kolmogorov-Smirnov test. Continuous variables with a normal distribution were presented as means ± standard error of the mean (SEM), while values without a normal distribution were presented as median (25%-75%). For comparisons between two groups, Student's t-test (two-tailed) or paired t-test was used. For multiple comparisons, one-way analysis of variance (ANOVA) was used followed by Tukey's post-hoc test. The Kruskal-Wallis test was used for non-normally distributed data. Categorical variables were presented as numbers and percentages, and analyzed using Fisher's exact test. Pearson's correlation analysis or Spearman rank correlation was used to investigate the correlation. Statistical significance was reported as follows: *P < 0.05, **P < 0.01, ***P < 0.001.

## Results

### CIH aggravates vascular injury and induces a pro-inflammatory phenotype of ECs

**Figure 1:**
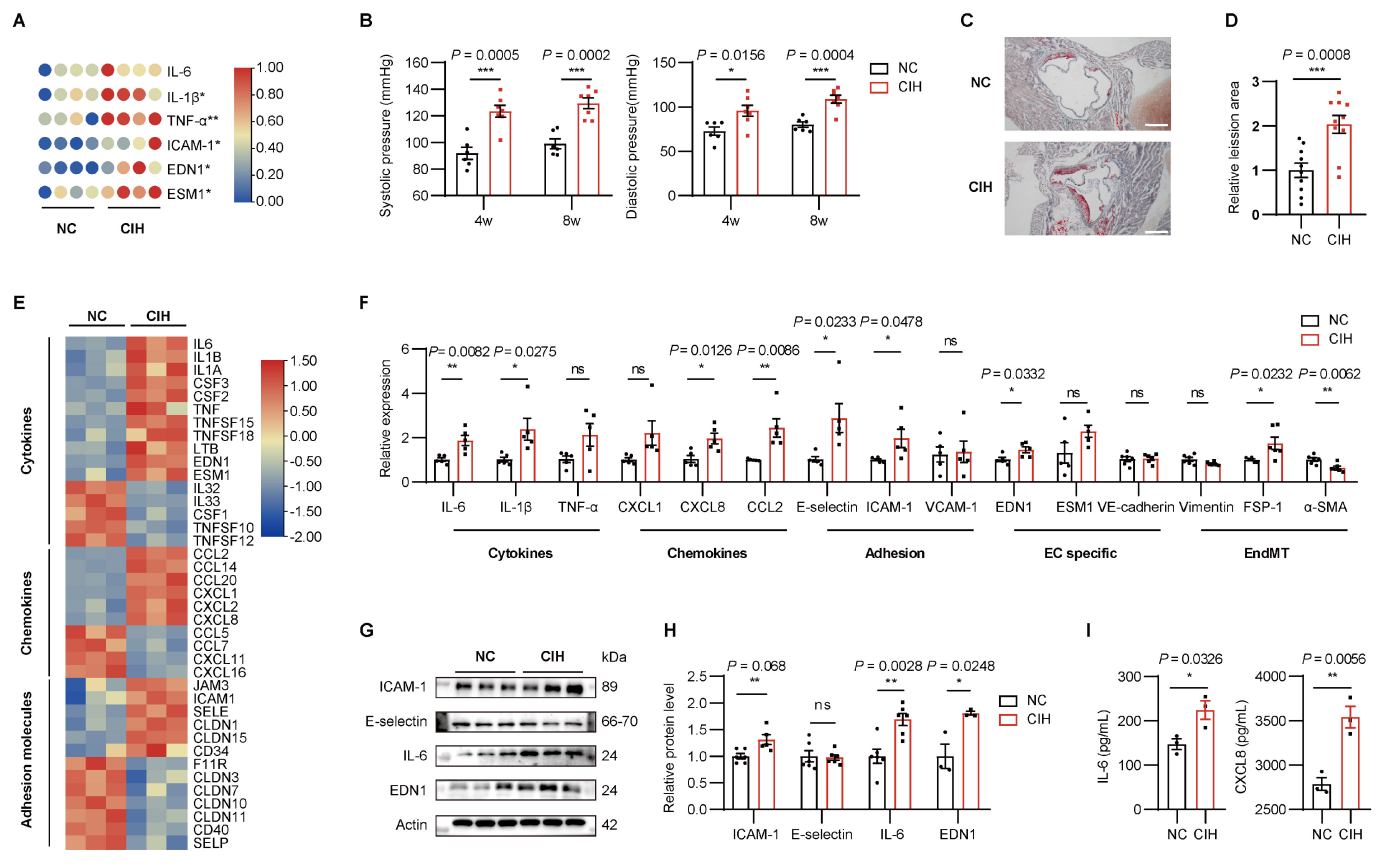
** CIH induces vascular endothelial injury. (A)** Heatmap representing normalized levels of inflammatory cytokines in APOE^-/-^ mice exposed to normoxia control (NC) or chronic intermittent hypoxia (CIH) for 8 weeks (n = 4 per group). Red and blue indicate high and low expression, respectively.** (B)** Systolic and diastolic blood pressure of APOE^-/-^ mice after 4 or 8 weeks of NC or CIH (n = 6-7 per group). **(C)** Representative cross-sectional images of the aortic root from APOE^-/-^ mice after 8 weeks of NC or CIH. Scale bar, 200 μm. **(D)** Quantitative analysis of atherosclerotic lesion area shown in (C) (n = 10 per group).** (E)** RNA-seq heatmap of differentially expressed genes (DEGs) related to cytokines, chemokines, and adhesion molecules in human umbilical vein endothelial cells (HUVECs) cultured under NC or CIH for 24 hours (n = 3 per group). Red and blue indicate up- and down-regulated genes, respectively. **(F)** Relative mRNA levels of representative cytokines (IL-6, IL-1β, and TNF-α), chemokines (CXCL1, CXCL8, and CCL2), adhesion molecules (E-selectin, ICAM-1, and VCAM-1), endothelial cell-specific molecules (EDN1, ESM1, and VE-cadherin), and endothelial-to-mesenchymal transition (EndMT) markers (Viementin, FSP-1, and α-SMA) in HUVECs under NC or CIH (n = 5 per group). **(G)** Representative Western blots of ICAM-1, E-selectin, IL-6, and EDN1. **(H)** Quantification of (G) (n = 6 per group). (**I**) Levels of IL-6 and CXCL8 in culture medium (n = 3 per group). Data are presented as mean ± SEM. Comparisons were performed by unpaired Student's t-test. Ns, no statistical significance, *P < 0.05, ** *P* < 0.01, *** *P* < 0.001.

Atherosclerosis-prone ApoE^-/-^ mice fed with a high-fat diet were exposed to normoxia control (NC) or CIH to investigate the effect of CIH^27^. CIH increased plasma levels of inflammatory cytokines and endothelial dysfunction markers, including IL-6, IL-1β, TNF-α, ICAM-1, EDN1, and ESM1, and induced increased blood pressure (Figure 1A-B, Supplementary Figure 1A). Given the critical role of leukocyte recruitment in atherosclerosis^28^, we observed that aortas from CIH mice contained more CD11b^+^ myeloid cells, particularly macrophages and inflammatory Ly6C^hi^ monocytes (Supplementary Figure 1B-C). Last, Oil Red O staining of aortic roots revealed increased atherosclerotic lesion area in the CIH group (Figure 1C-D). Together these data demonstrated that CIH exacerbated vascular injury, characterized by systemic inflammation, increased blood pressure, promoting the infiltration of myeloid subsets into the aortas and aggravating atherosclerosis.

To comprehensively assess CIH-induced transcriptional alterations in ECs, we performed RNA sequencing (RNA-seq) in human umbilical vein endothelial cells (HUVECs) treated with either NC or CIH. CIH up-regulated 11 out of 16 cytokines, 6 out of 10 chemokines, and 6 out of 13 adhesion molecules, suggesting the inflammatory signature of HUVECs under CIH (Figure 1E). Validation of above key molecules by RT-qPCR confirmed that CIH significantly upregulated the expression of IL-6, IL-1β, CXCL8, CCL2, E-selectin, ICAM-1, and EDN1 (Figure 1F). Western blot (WB) analysis further verified the increased protein levels of ICAM-1, IL-6, and EDN1 (Figure 1G-H). And secretion of IL-6 and CXCL8 increased in the CIH group (Figure 1I). Taken together, CIH induced a pro-inflammatory phenotype of ECs, characterized by upregulated expression of cytokines, chemokines, and adhesion molecules, favoring the vascular injury.

### CIH induces imbalanced glycolytic/mitochondrial metabolism of ECs

**Figure 2:**
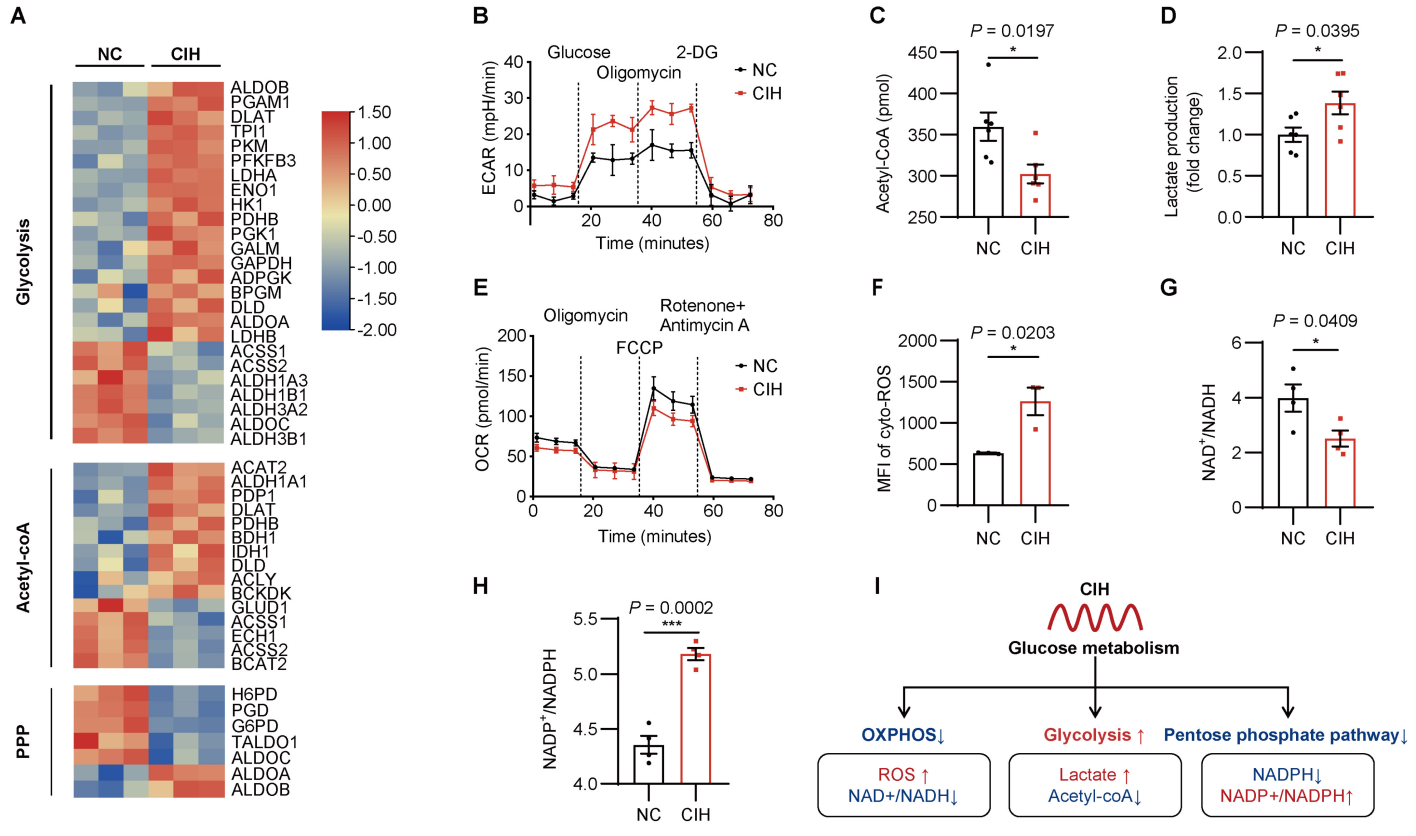
** Rewiring of glucose metabolism in ECs under CIH.** HUVECs were cultured under NC or CIH. **(A)** Heatmap of DEGs involved in glycolysis, generation of Acetyl-coA, and the pentose phosphate pathway (PPP). **(B)** Glycolytic flux measured by extracellular acidification rate (ECAR) (n = 8 per group). (**C, D**) Cellular levels of acetyl-CoA and lactate (n = 6 per group).** (E)** Mitochondrial respiration assessed by oxygen consumption rate (OCR) (n = 10 per group).** (F)** Flow cytometric analysis of cytoplasmic reactive oxygen species (cyto-ROS) (n = 3 per group).** (G, H)** Cellular NAD^+^/ NADH and NADP^+^/ NADPH (n = 4 per group).** (I)** Schematic of metabolic reprogramming for glucose flux regulated by CIH. OXPHOS, oxidative phosphorylation. Data are presented as mean ± SEM. Comparisons were performed by unpaired Student's t-test. Ns, no statistical significance, *P < 0.05, ** *P* < 0.01, *** *P* < 0.001.

Metabolic derangement is a key culprit in vascular pathophysiology. KEGG (Kyoto Encyclopedia of Genes and Genomes) pathway analysis of RNA-seq data revealed that carbon metabolism ranked among the top enriched pathways (Supplementary Figure 2A), we then investigated how metabolic state of ECs was rewired by CIH by performing RNA-seq analysis of glucose metabolic enzymes (Figure 2A). As ECs are glycolysis addicted, CIH significantly up-regulated 18 out of the 71 glycolytic enzymes (Figure 2A). Seahorse flux analysis further demonstrated an increase in both glycolysis and glycolytic capacity following CIH stimulation (Figure 2B, Supplementary Figure 2B), coinciding with decreased acetyl-CoA levels and increased lactate production (Figure 2C-D).

Though mitochondria compose only 2-6% of the cell volume, mitochondria exert profound influence on ECs function through regulation of reactive oxygen species (ROS) generation and nicotinamide adenine dinucleotide (NAD^+^)/NADH redox balance^29^. In contrast to enhanced glycolysis, CIH impaired mitochondrial respiration, evidenced by reduced oxygen consumption rate (OCR) (Figure 2E, Supplementary Figure 2C). Enhanced production of ROS, decreased ratio of NAD^+^/NADH, and breakdown of mitochondrial membrane potential were observed under CIH (Figure 2F-G, Supplementary Figure 2D-E), suggesting mitochondrial dysfunction and oxidative stress injury.

Since glycolysis and pentose phosphate pathway (PPP) pathway both competitively consume glucose-6-phosphate (G-6-P) as metabolic substrate, the metabolic flux of PPP pathway was also influenced. RNA-seq analysis revealed that CIH significantly down-regulated 5 PPP-related enzymes, including the key rate-limiting enzyme glucose-6-phosphate dehydrogenase (G6PD) (Figure 2A). Consistently, CIH reduced intracellular nicotinamide adenine dinucleotide phosphate (NADPH) content and increased the ratio of NADP^+^/NADPH (Figure 2H, Supplementary Figure 2F). These results collectively delineated the metabolic reprogramming of ECs under CIH, characterized by enhanced glycolysis, reduced mitochondrial respiration, and impaired PPP activity (Figure 2I).

### PFKFB3 is markedly increased in ECs under CIH

**Figure 3:**
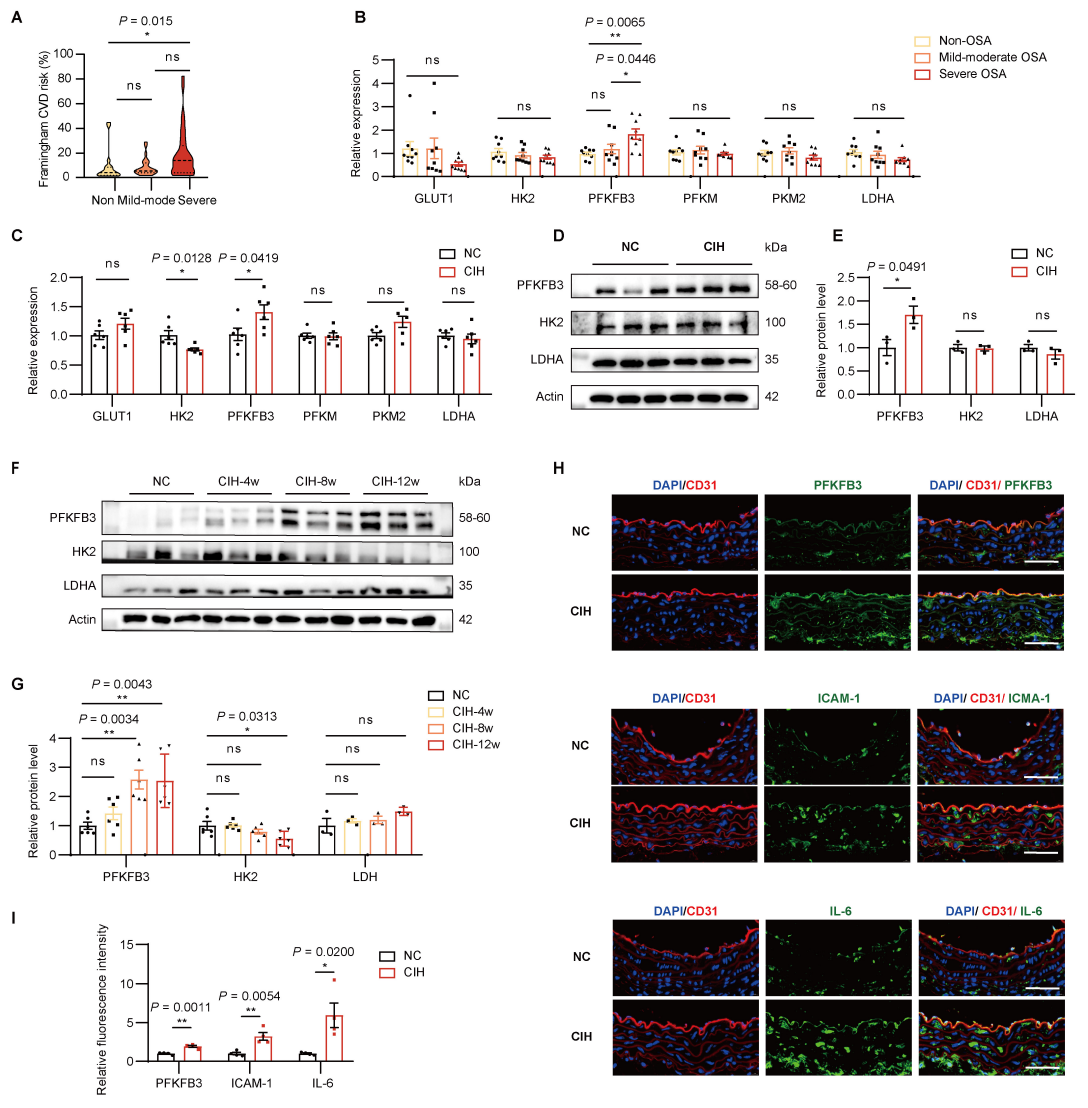
** CIH upregulates PFKFB3 expression. (A)** Framingham cardiovascular diseases (CVD) risk among non-obstructive sleep apnea (OSA), mild-moderate (mild-mode) OSA, and severe OSA groups.** (B)** Relative mRNA levels of key glycolytic enzymes including GLUT1, HK2, PFKFB3, PFKM, PKM2, and LDHA in HUVECs treated with 20% plasma from non-OSA, mild to moderate OSA, or severe OSA (n = 9 per group).** (C)** Relative mRNA levels of key glycolytic enzymes in HUVECs under NC or CIH (n = 6 per group).** (D)** Representative Western blots of PFKFB3, HK2, and LDHA in HUVECs under NC or CIH.** (E)** Quantification of (D**)** (n = 3 per group).** (F)** Representative Western blots of PFKFB3, HK2, and LDHA of aortas from C57BL/6J mice of NC, CIH-4w, CIH-8w, and CIH-12w.** (G)** Quantification of (F**)** (n = 3-6 per group).** (H)** Representative immunofluorescence images of aortas from C57BL/6J mice exposed to NC or CIH for 8weeks. Sections were co-stained for PFKFB3, ICAM-1, or IL-6 (green), CD31 (red) and DAPI (blue). Scale bar, 100 μm.** (I)** Quantification of (H**)** (n = 4 per group). The data are presented as mean ± SEM. Statistical significance was determined by unpaired Student's t-test (**C, E, I**), Kruskal-Wallis test (**A**), or ANOVA (**B, G**). Ns, no statistical significance, *P < 0.05, ** *P* < 0.01, *** *P* < 0.001.

Participants with suspected OSA were recruited and underwent anthropometric measurements, overnight polysomnography, and blood sample collection (Supplementary Table.1). The severity of OSA was significantly associated with an increased 10-year cardiovascular risk as assessed by the Framingham CVD risk algorithm (Figure 3A)^22^. Given the essential role of glycolysis in ECs, we examined the expression of key enzymes in glycolytic pathways. In *ex vivo* incubation experiments using plasma samples of healthy control or patients with OSA, who were age, sex and body mass index matched, we observed significantly elevated PFKFB3 transcript levels in the severe OSA group (Figure 3B). And the concentration of PFKFB3 tended to increase follow the severity of OSA (Supplementary Figure 3A). Similarly, *in vitro* CIH treatment up-regulated the transcript and protein levels of PFKFB3 (Figure 2 C-E, Supplementary Figure 3C).

Consistent with these findings, PFKFB3 protein levels from aortas of mice significantly increased in CIH-8w group, which remained similar in CIH-12w group, while the levels of HK2 showed a downward trajectory (Figure 3F-G). And a higher secretion of PFKFB3 was also observed in plasma of mice under CIH (Supplementary Figure 3D).

Since lactate levels were elevated in the CIH group, we investigated whether lactate dehydrogenase (LDH), the key enzyme regulating lactate synthesis, was involved. However, LDH expression remained unchanged under CIH (Figure 2B-G, Supplementary Figure 3B). To further determine whether increased PFKFB3 expression was mainly attributed to the endothelial layer, we performed double immunofluorescence staining of aortic sections using a well-characterized monoclonal antibody for PFKFB3 and the ECs marker CD31. 8 weeks of CIH markedly increased PFKFB3 expression in the aortic endothelium, coinciding with elevated ICAM-1 and IL-6 levels (Figure 3H-I). Altogether, these results indicated that CIH induced elevated levels of PFKFB3 *in vivo* and *in vitro*.

### PFKFB3 mediated glycolysis drives CIH induced endothelial inflammation

To determine the role of PFKFB3 as a glycolytic activator, over-expression of PFKFB3 expectedly potentiated glycolysis and suppressed mitochondrial respiration (Supplementary Figure 4A-C), further supporting the imbalanced glycolytic/mitochondrial metabolism under CIH.

**Figure 4:**
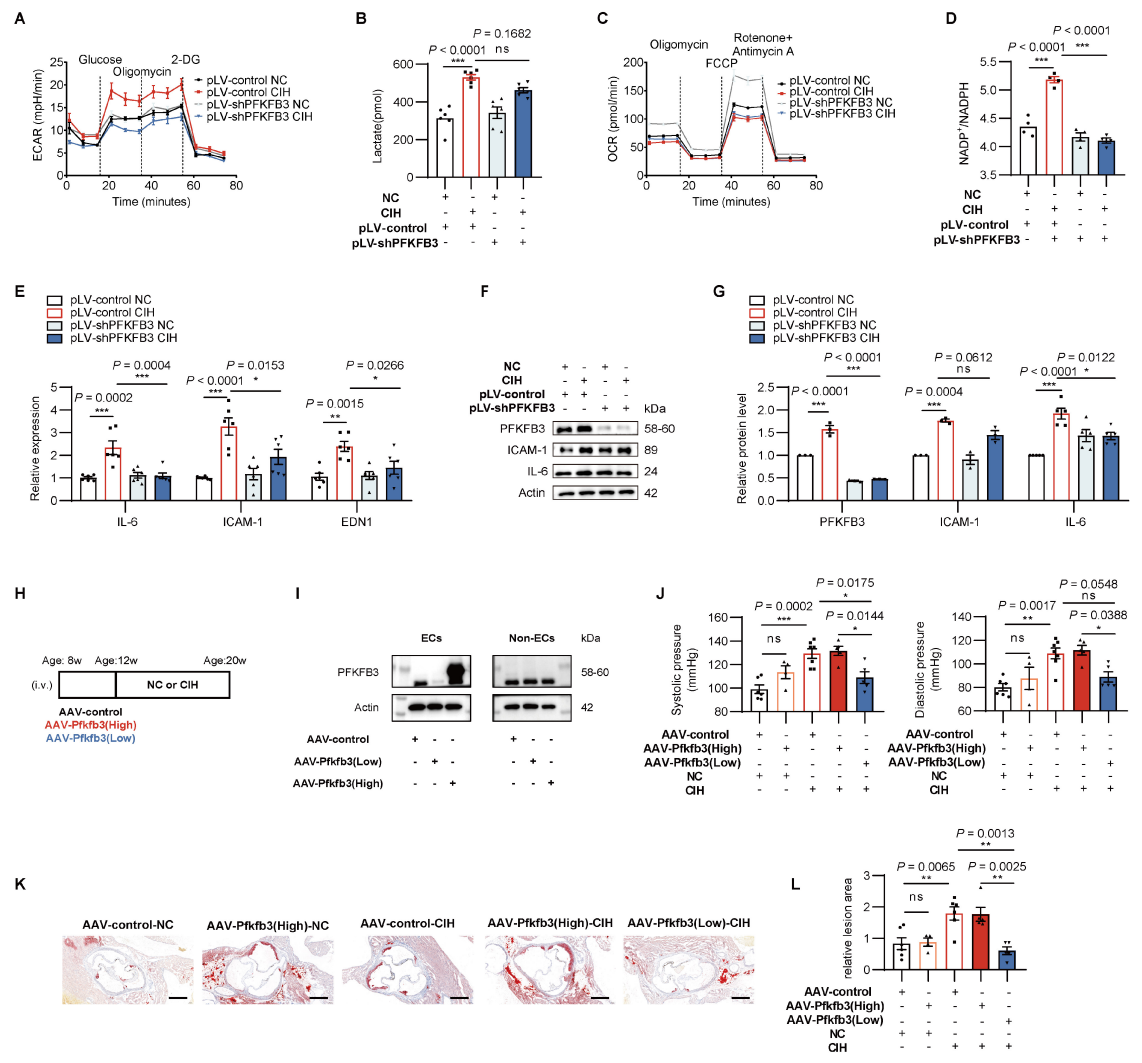
** PFKFB3 mediated glycolysis drives CIH induced endothelial inflammation and vascular injury.** HUVECs were transfected with either pLV-shPFKFB3 or pLV-control and cultured under NC or CIH.** (A)** Glycolytic flux measured by ECAR (n = 6-8 per group).** (B)** Cellular lactate levels (n = 6 per group).** (C)** Mitochondrial respiration assessed by OCR (n = 6-8 per group).** (D)** Cellular NADP^+^/ NADPH ratio (n = 4 per group). **(E)** Relative mRNA levels of IL-6, ICAM-1, and EDN1 (n = 6 per group).** (F)** Representative Western blots of PFKFB3, ICAM-1, and IL-6.** (G)** Quantification of **(**F**)** (n = 3-5 per group).** (H)** Schematic for AAV-based endothelial-specific over-expression or knockdown of Pfkfb3 followed by NC or CIH in APOE^-/-^ mice. **(I)** Representative Western blots of PFKFB3 in aortic ECs and remaining tissues (Non-ECs) isolated from AAV-control, AAV-Pfkfb3(Low), and AAV-Pfkfb3(High) mice. **(J)** Systolic and diastolic blood pressure (n = 4-6 per group).** (K)** Representative cross-sectional images of the aortic root. Scale bar, 400 μm. **(L)** Quantitative of atherosclerotic lesion area in (K) (n = 4-6 per group). The data are presented as mean ± SEM. Statistical significance was determined by ANOVA. Ns, no statistical significance, *P < 0.05, ** *P* < 0.01, *** *P* < 0.001.

To determine whether PFKFB3 drives glycolytic reprogramming and endothelial inflammation under CIH, we introduced pLV-shPFKFB3 into HUVECs to silence PFKFB3 expression (Supplementary Figure 4D). PFKFB3 knockdown restored the imbalanced glycolytic/mitochondrial metabolism and impaired PPP (Figure 4A-D, Supplementary Figure 4E-F). Silencing of PFKFB3 further diminished the enhancive effect of CIH on transcription of IL-6, ICAM-1, and EDN1 (Figure 4E), as well as protein levels of ICAM-1 and IL-6 (Figure 4F-G).

To gain insight into whether endothelial PFKFB3 plays a causal role in CIH-induced vascular injury *in vivo*, we introduced vascular endothelial-specific Pfkfb3-over-expression (AAV-Pfkfb3(High)) or Pfkfb3-knockdown (AAV-Pfkfb3(Low)) mice via intravenous injection of adeno-associated virus (AAV) (Figure 4H)^18^. Vascular specific regulation was visualized by living imaging and endothelial specific expression was visualized by immunofluorescent staining in CD31-positive vascular cells (Supplementary Figure 5A-B). Western blot analysis of primary aortic ECs and non-ECs isolated from AAV-treated mice demonstrated selective alteration of PFKFB3 expression in ECs (Figure 4I).

AAV-Pfkfb3(Low)-CIH mice reversed the increased blood pressure compared to AAV-control-CIH in C57BL/6J models (Supplementary Figure 5C). In APOE^-/-^ models, a similar decrease in blood pressure was observed in AAV- Pfkfb3(Low)-CIH mice compared with AAV-control-CIH group or AAV- Pfkfb3(High)-CIH group (Figure 4J). In addition, endothelial-specific Pfkfb3 silencing suppressed CIH induced formation of atherosclerotic lesions (Figure 4K-L), indicating the vital role of PFKFB3 in mediating CIH induced endothelial inflammation and vascular injury.

### Inhibition of PFKFB3 alleviates CIH induced endothelial inflammation and vascular injury

We next asked whether pharmacological intervention targeting PFKFB3 could prevent endothelial inflammation and vascular injury under CIH. To this end, 3-(3-pyridinyl)-1-(4-pyridinyl)-2-propen-1-one (3PO), the specific PFKFB3 inhibitor^30^, ^31^, or Salvianolic acid C (SAC), a phenolic component derived from Salvia miltiorrhiza (Danshen), which has been proven to suppress PFKFB3 via accelerating its degradation^18^, was administered before CIH challenge.

**Figure 5:**
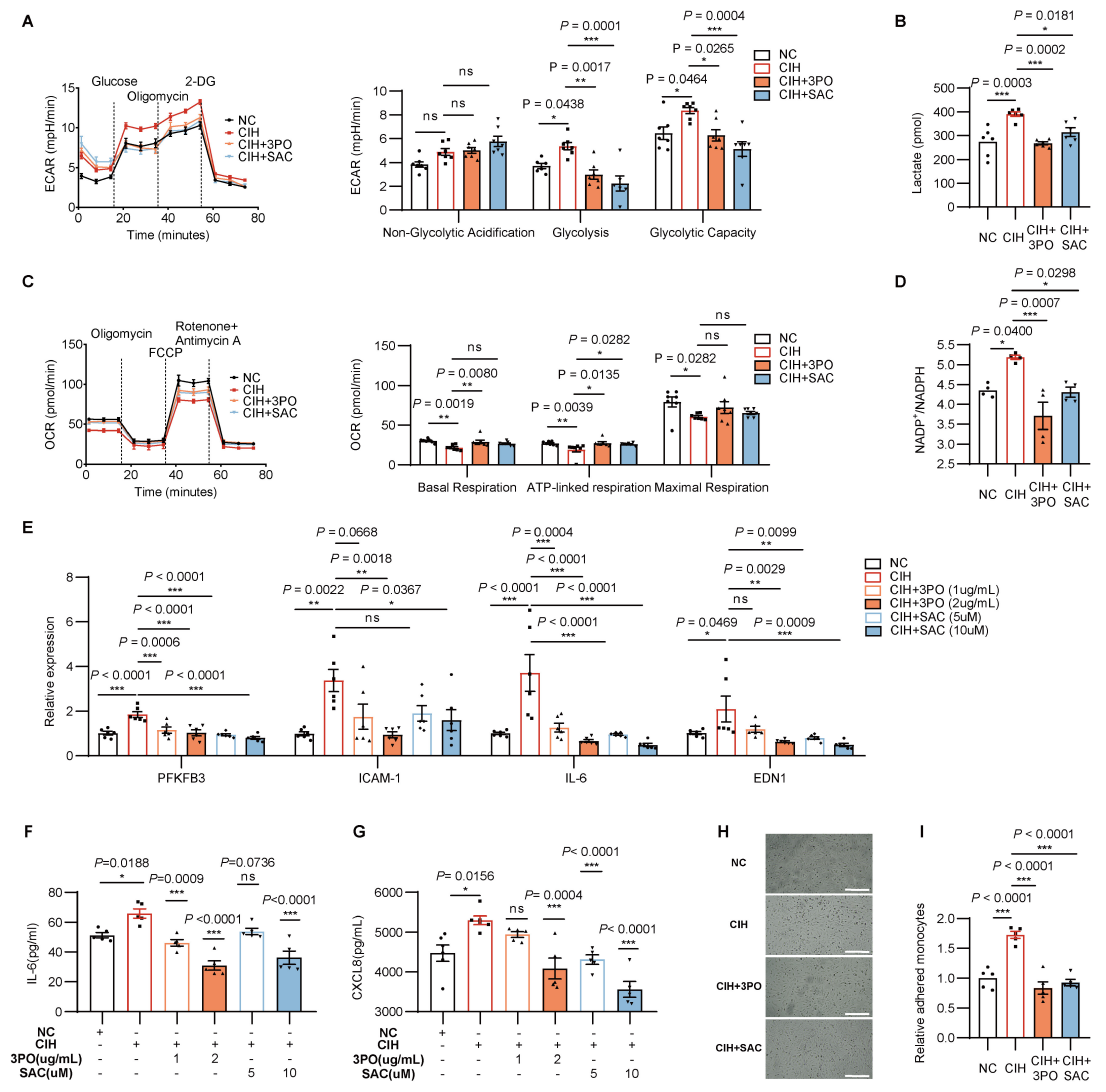
** Pharmacological inhibition of PFKFB3 restores CIH induced metabolic imbalance and endothelial inflammation.** HUVECs were treated with NC, CIH, CIH added with 3PO, or CIH added with SAC. **(A)** Glycolytic flux measured by ECAR (n = 7 per group).** (B)** Cellular lactate levels (n = 6 per group).** (C)** Mitochondrial respiration assessed by OCR (n = 7 per group).** (D)** Cellular NADP^+^/ NADPH ratio (n = 4 per group).** (E)** Relative mRNA levels of PFKFB3, ICAM-1, IL-6, and EDN1 (n = 6 per group).** (F, G)** Levels of IL-6 and CXCL8 in culture medium (n = 5 per group). **(H)** Representative images of monocytes adhered to HUVECs. Adhered monocytes were visualized as dark round cells. Scale bar, 20 μm.** (I)** Quantification of adhered monocytes in (H) (n = 5 per group). The data are presented as mean ± SEM. Statistical significance was determined by ANOVA. Statistical significance is indicated for comparisons with the CIH group. Ns, no statistical significance, *P < 0.05, ** *P* < 0.01, *** *P* < 0.001.

In aspects to metabolic changes under CIH, Seahorse flux analysis showed that either 3PO or SAC administration repressed the increase of glycolysis caused by CIH, accompanied by decreased intracellular lactate level (Figure 5A-B). Additionally, either 3PO or SAC treatment prevented the reduction of mitochondrial respiration (Figure 5C) and contributed to decreased cellular NADP^+^/NADPH ratio (Figure 5D), suggesting restoration of glycolytic homeostasis.

**Figure 6:**
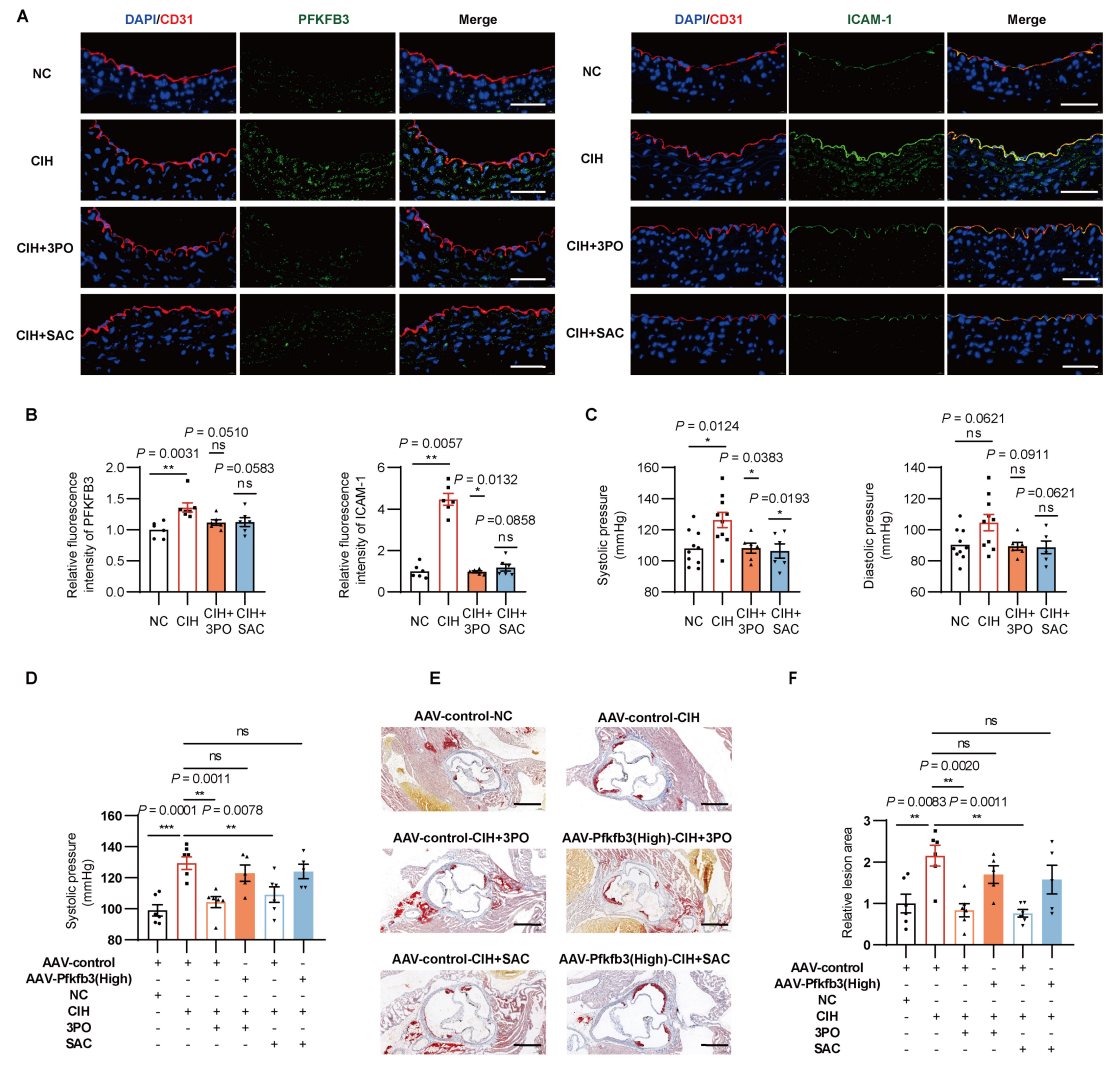
** Pharmacological inhibition of PFKFB3 alleviates CIH induced vascular injury. (A)** Representative immunofluorescence images of aortas stained for PFKFB3 or ICAM-1 (green), CD31 (red) and DAPI (blue). Scale bar, 100 μm.** (B)** Quantification of fluorescence intensity in **(**A**)** (n = 6-7 per group). **(C)** Systolic and diastolic blood pressure in C57BL/6J mice exposed to NC, CIH, or CIH injected with 3PO or SAC (n = 6-10 per group). **(D)** Systolic blood pressure of APOE^-/-^ mice grouped by AAV-control-NC, AAV-control-CIH, AAV-control-CIH+3PO, AAV-Pfkfb3(high)-CIH+3PO, AAV-control-CIH+SAC, or AAV-Pfkfb3(high)-CIH+SAC (n = 5-7 per group).** (E)** Representative Oil Red O-stained cross-sections of the aortic root. Representative images for the AAV-control-NC and AAV-control-CIH groups are shared with Figure 4K, as all groups were processed in the same experiment. Scale bar, 400 μm.** (F)** Quantitative analysis of (E) (n = 5-7 per group). The data are presented as mean ± SEM. Statistical significance was determined by ANOVA. Statistical significance was indicated for comparisons with the CIH group. Ns, no statistical significance, *P < 0.05, ** *P* < 0.01, *** *P* < 0.001.

Regarding the endothelial inflammation, both inhibitors suppressed the up-regulated transcripts of inflammatory markers including IL-6, ICAM-1, and EDN1 (Figure 5E), and reduced secretion of IL-6 and CXCL8 (Figure 5F-G). Consistent with these findings, 3PO or SAC also repressed monocytes adhesion promoted by CIH (Figure 5H-I). *In vivo*, immunofluorescent counterstaining demonstrated decreased PFKFB3 or ICAM-1 intensity in vascular ECs following 3PO or SAC treatment (Figure 6A-B). Concordantly, heightened blood pressure induced by CIH exposure was attenuated after 3PO or SAC treatment (Figure 6C), demonstrating the therapeutic potential in mitigating endothelial dysfunction and vascular pathology.

To further examine the essential role of PFKFB3, we introduced AAV-control or AAV-Pfkfb3(High) to APOE^-/-^ models. Pharmacological inhibition of PFKFB3 by 3PO or SAC ameliorated CIH induced high blood pressure (Figure 6D, Supplementary Figure 6A), CD11b^+^ myeloid cells and inflammatory monocytes infiltration (Supplementary Figure 6B), and formation of atherosclerotic lesions (Figure 6E-F). However, endothelial-specific Pfkfb3 over-expression diminished those protective effects (Figure 6D-F, Supplementary Figure 6). Above results collectively supported the therapeutic potential of PFKFB3 inhibition to combat CIH associated inflammatory responses and vascular injury.

### The HIF-1α/PFKFB3 axis mediates the glycolysis reprogramming and vascular endothelial injury

**Figure 7:**
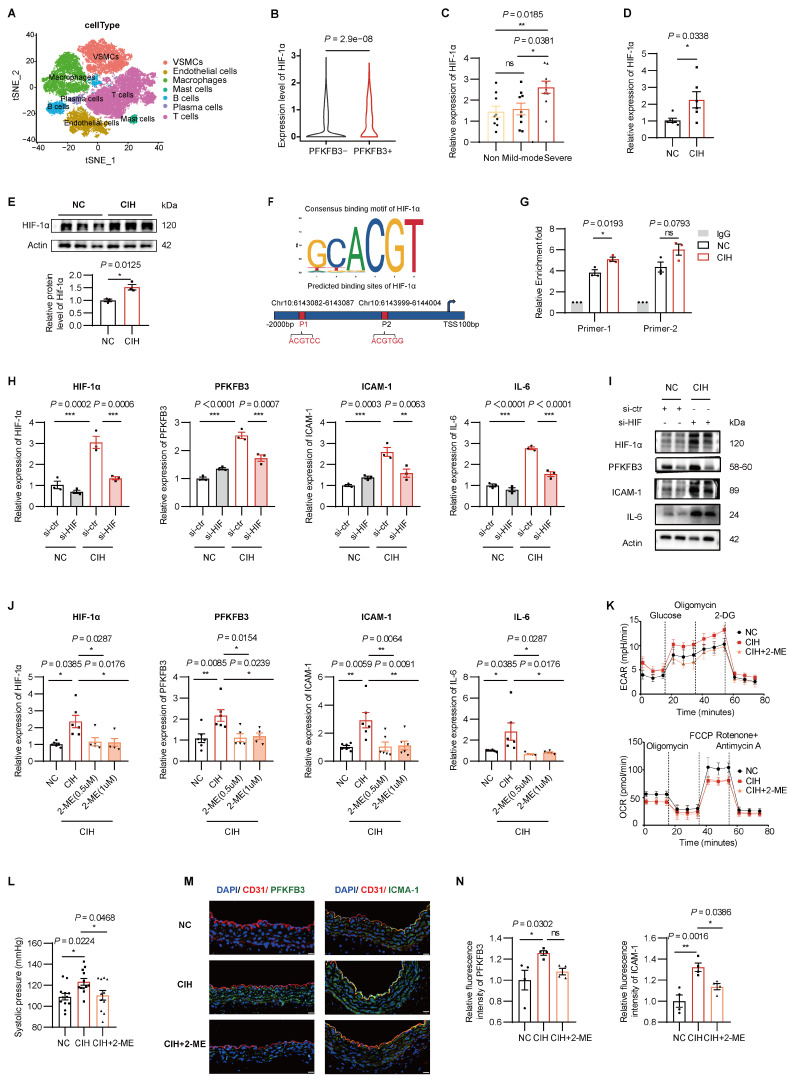
** CIH activates HIF-1α which regulates the expression of PFKFB3. (A)** T-distributed stochastic neighbor embedding (t-SNE) visualization of major cell types from single-cell RNA-seq (scRNA-seq) analysis based on carotid artery tissue.** (B)** Expression of HIF-1α in PFKFB3-high ECs (PFKFB3+) versus PFKFB3-low ECs (PFKFB3-) from scRNA-seq.** (C)** Relative mRNA levels of HIF-1α in HUVECs treated with 20% plasma from non-OSA, mild to moderate OSA, or severe OSA (n = 9-10 per group). **(D)** Relative mRNA levels of HIF-1α in HUVECs under NC or CIH (n = 6 per group).** (E)** Western blots of HIF-1α in HUVECs under NC or CIH (n = 3 per group). **(F)** Consensus binding motif of HIF-1α and the binding sites of HIF-1α on the promoter of PFKFB3 from JASPAR database. **(G)** CUT&Tag-qPCR analysis of the indicated promoters using antibodies against HIF-1α (n = 3 per group). **(H)** Relative mRNA levels of HIF-1α, PFKFB3, ICAM-1, and IL-6 in HUVECs transfected with si-control (si-ctr) or si-HIF-1α (si- HIF) under NC or CIH (n = 3 per group). **(I)** Representative Western blots of HIF-1α, PFKFB3, ICAM-1, and IL-6 in HUVECs transfected with si-ctr or si- HIF under NC or CIH. **(J)** Relative mRNA levels of HIF-1α, PFKFB3, ICAM-1, and IL-6 in HUVECs under NC, CIH, or CIH + 2-ME (n = 5-6 per group). **(K)** Glycolytic flux (ECAR) and mitochondrial respiration (OCR) in HUVECs under NC, CIH, or CIH + 2-ME (n = 7 per group). **(L)** Systolic blood pressure in C57BL/6J mice exposed to NC, CIH, or CIH+2-ME (n = 6-10 per group). **(M)** Representative immunofluorescence of aortas derived from C57BL/6J mice exposed to NC, CIH, or CIH + 2-ME. Sections were co-stained for PFKFB3 or ICAM-1 (green), CD31 (red), and DAPI (blue). Scale bar, 20 μm.** (N)** Quantification of (M) (n = 4 per group). The data are presented as mean ± SEM. Statistical significance was determined by unpaired Student's t test (B, D, E, G) or ANOVA (C, H, J, L, N). Ns, no statistical significance, *P < 0.05, ** *P* < 0.01, *** *P* < 0.001.

Due to the presence of multiple copies of the AUUUA instability motif in its 3' untranslated region, PFKFB3 is susceptible to upstream regulation^30^, ^32^. Prior studies have indicated potential regulation by HIF, RAS, mTOR, or YAP-TEAD1 signaling^20^. We therefore examined those regulators in HUVECs treated with plasma stimulation models or CIH exposure (Supplementary Figure 7A-B). Bioinformatic analysis using Gene Expression Profiling Interactive Analysis (GEPIA) database (http://gepia.cancer-pku.cn/) revealed significant positive correlation between PFKFB3 and HIF-1α (r = 0.4, *P* = 3.6e-9) rather than HIF-2α (r = -0.092, *P* = 0.19) (Supplementary Figure 7C). In the context of atherosclerosis, single-cell RNA-seq analysis of carotid atherosclerotic plaques (GSE159677) further demonstrated higher HIF-1α expression in PFKFB3-high ECs versus PFKFB3-low ECs (*P* = 2.9×10-8) (Figure 7A-B, Supplementary Figure 7D-E)^24^. And the increased levels of HIF-1α in response to plasma stimulation or CIH were further validated (Figure 7C-E).

We therefore investigated the regulated role of HIF-1α on PFKFB3. Using JASPAR (http://jaspar.genereg.net/) and UCSC (https://genome.ucsc.edu/), we predicted two putative HIF-1α binding sites within the promoter region of PFKFB3 (Figure 7F). CUT&Tag-qPCR confirmed enhanced HIF-1α binding on the promoters of PFKFB3 in HUVECs exposed to CIH (Figure 7G).

To investigate the role of HIF-1α activation, HIF-1α knockdown by siRNA significantly reduced CIH-induced upregulation of PFKFB3, ICAM-1, and IL-6 (Figure 7H-I). Consistently, pharmacological inhibition of HIF-1α activity by 2-methoxyestradiol (2-ME) significantly attenuated CIH-induced PFKFB3 upregulation, restored the imbalanced glycolytic/mitochondrial metabolism, and suppressed proinflammatory responses triggered by CIH *in vitro* (Figure 7J-K, Supplementary Figure 8). *In vivo*, 2-ME abolished the effects of CIH on elevated systolic pressure and heightened expression of ICAM-1 intensity in vascular ECs (Figure 7L-N).

**Figure 8:**
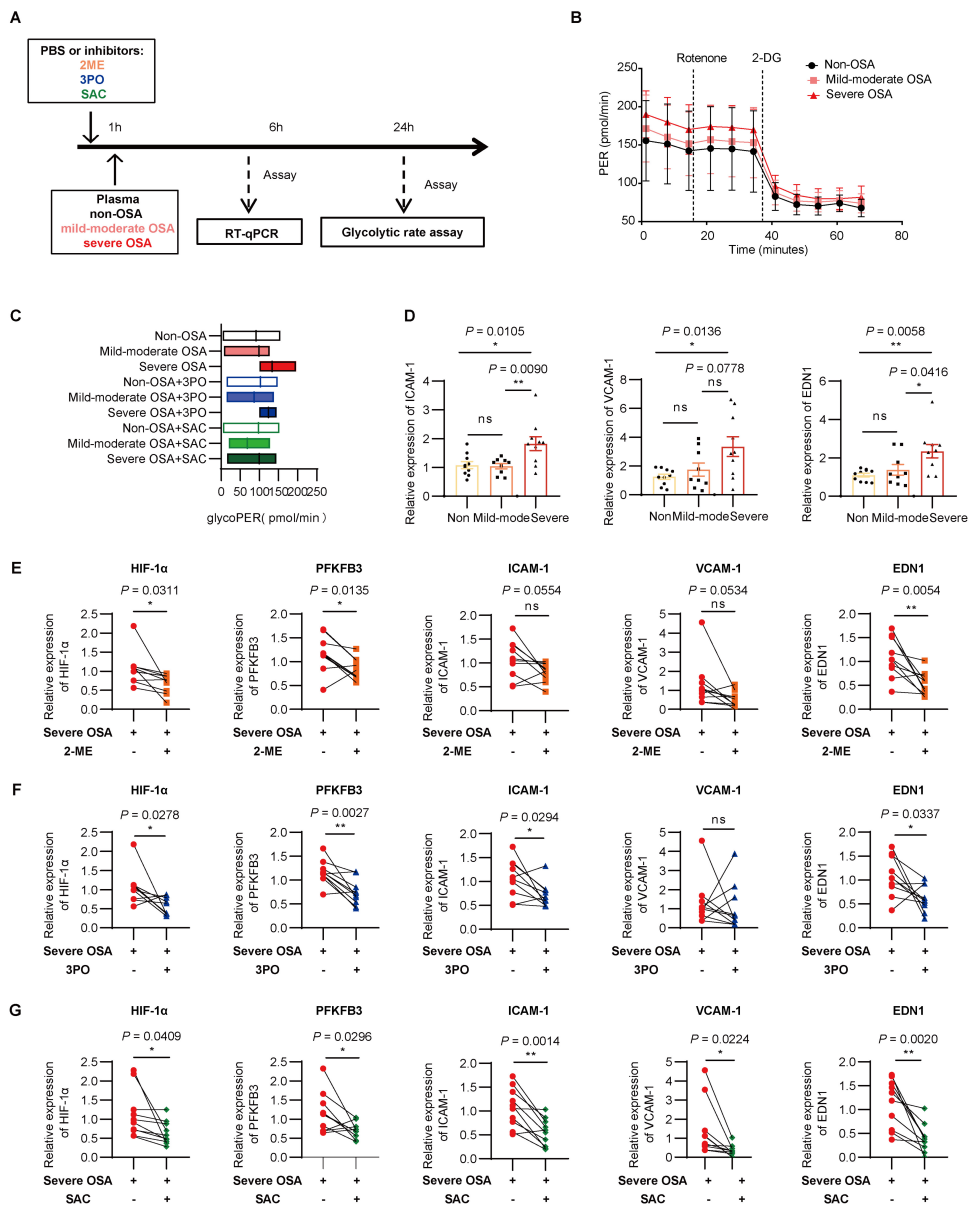
**Targeting the HIF-1α/PFKFB3 axis alleviates OSA induced endothelial inflammation and glycolysis. (A)** Schematic representation of the plasma stimulation model. **(B)** Glycolytic rate assay of HUVECs treated with 20% plasma from non-OSA, mild to moderate OSA, and severe OSA (n = 5-6 per group). **(C)** Glycolytic proton efflux rate (PER) of each group (n = 5-6 per group).** (D)** Relative mRNA levels of ICAM-1, VCAM-1, and EDN1 in HUVECs treated with plasma from each OSA group (n = 9-10 per group). **(E-G)** Relative mRNA levels of HIF-1α, PFKFB3, ICAM-1, VCAM-1, and EDN1 in HUVECs treated with severe OSA plasma alone or with **(E)** 2-ME, **(F)** 3PO, or **(G)** SAC (n = 10 per group). The data are presented as mean ± SEM. Statistical significance was determined by ANOVA (**D**) or paired t-test (**F-G**). Ns, no statistical significance, *P < 0.05, ** *P* < 0.01, *** *P* < 0.001.

Lastly, to validate the therapeutic potential for pharmacological intervention of HIF-1α/PFKFB3 axis, we performed an in vitro model using plasma samples of non-OSA, mild to moderate OSA, or severe OSA participants, with inhibitors including 2-ME, 3PO, or SAC (Figure 8A). Seahorse flux analysis revealed an increase of endothelial glycolysis following the OSA severity, which were reduced by each of the three inhibitors (Figure 8B-C, Supplementary Figure 9A). Along this same line, as increased transcription of ICAM-1, VCAM-1, and EDN1 were observed in severe OSA group compared with non-OSA group (Figure 8D, Supplementary Figure 9B). Lactate alone, at concentrations matching OSA plasma, partially induced these effects but was less potent than plasma stimulation (Supplementary Figures 9C-D). 2-ME, 3PO, or SAC could alleviate the expression of these inflammatory mediators in severe OSA (Figure 8E-G). These data suggest that endothelial glycolysis and inflammation activation induced by OSA could be suppressed by pharmacological intervention targeting the HIF-1α/PFKFB3 axis.

### PFKFB3-driven lactate promotes H3K18 lactylation, forming a PFKFB3-H3K18la feedback loop

**Figure 9:**
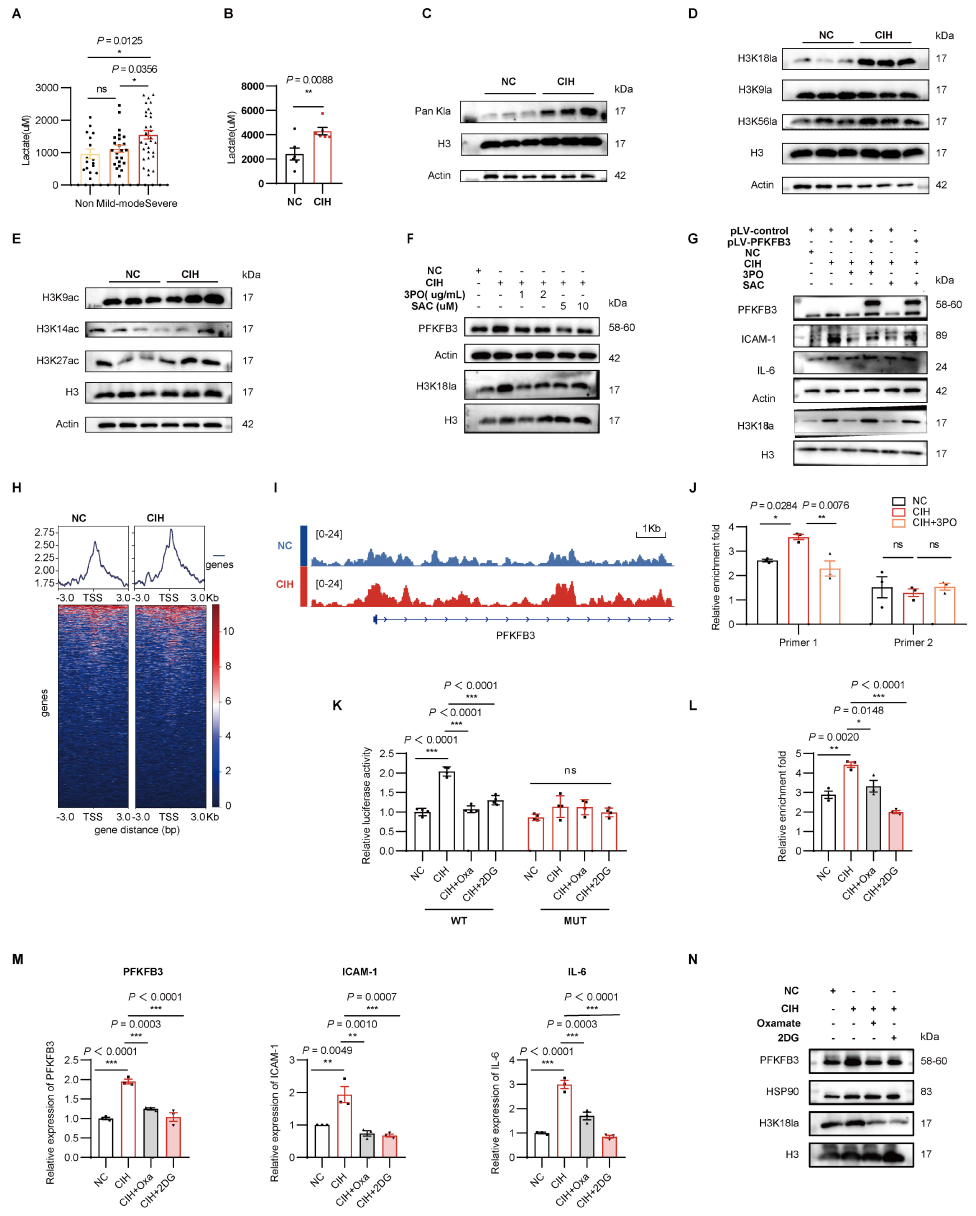
** A PFKFB3-lactate-H3K18la positive feedback loop in CIH. (A)** Plasma lactate levels across OSA groups.** (B)** Plasma lactate in C57BL/6J mice under NC or CIH (n = 6 per group).** (C-E)** Representative Western blotting analysis of **(C)** pan-histone lactylation (Pan Kla), **(D)** site-specific histone lactylation, and **(E)** site-specific histone acetylation in HUVECs under NC or CIH.** (F)** Representative Western blots of H3K18la in HUVECs treated with NC, CIH, or CIH added with 3PO or SAC. **(G)** Representative Western blots of PFKFB3, ICAM-1, IL-6, and H3K18la in HUVECs transfected with either pLV-control or pLV-PFKFB3 followed by NC, CIH, CIH + 3PO, or CIH + SAC.** (H)** Binding density of H3K18la visualized by deepTools. The heatmap presents the CUT&Tag tag counts on the H3K18la binding peaks in chromosome 10.** (I)** Genome browser tracks of CUT&Tag signal at the representative target gene loci. Red rectangles indicate the peak regions of H3K18la on target-gene promoters. **(J)** CUT&Tag-qPCR analysis of the indicated promoters of PFKFB3 using antibodies against H3K18la in HUVECs under NC, CIH, or CIH + 3PO (n = 3 per group). **(K)** Dual-luciferase reporter assay of wild-type (WT) or mutant (MUT) PFKFB3 promoter activity in HUVECs under NC, CIH, CIH + Oxamate (CIH+Oxa), or CIH + 2DG (n = 3 per group). **(L)** CUT&Tag-qPCR analysis of the indicated promoters of PFKFB3 using antibodies against H3K18la in HUVECs under NC, CIH, CIH+Oxa, or CIH + 2DG (n = 3 per group). **(M)** Relative mRNA levels of PFKFB3, ICAM-1, and IL-6 in HUVECs under NC, CIH, CIH+Oxa, or CIH + 2DG (n = 3 per group). **(N)** Representative Western blots of PFKFB3 and H3K18la in HUVECs under NC, CIH, CIH+Oxa, or CIH + 2DG. The data are presented as mean ± SEM. Statistical significance was determined by unpaired Student's t tes** (B)** or ANOVA** (A, J-M)**. Ns, no statistical significance, *P < 0.05, ** *P* < 0.01, *** *P* < 0.001.

Endothelial glucose metabolism predominately switched to anaerobic metabolism under CIH, resulting in elevated concentration of lactate and decreased cellular acetyl-CoA levels (Figure 2C-D). Elevated lactate concentration was found in severe OSA patients compared with non-OSA group (Figure 9A), which was also observed in plasma of C57BL/6J mice subjected to CIH (Figure 9B). Both lactate and acetyl-CoA are central precursors that have major impact on protein lactylation^33^ or acetylation^34^, respectively, especially histone modification. Thus, we performed WB analysis with antibodies against diverse forms of histone lactylation or acetylation, and observed an increase in the levels of both Pan-lysine lactylation (Pan Kla) and H3K18la (Figure 9C-E). H3K18la protein levels were also markedly increased in aortas of mice subjected to CIH (Supplementary Figure 10A-B). Besides, inhibition of PFKFB3 by 3PO or SAC ameliorated CIH induced high level of H3K18la (Figure 9F). PFKFB3 over-expression reversed the suppressive effects of 3PO and SAC on CIH-induced upregulation of H3K18la and inflammatory markers (Figure 9G). These results indicate that histone lactylation was increased in the context of CIH, and H3K18la was the most prevalent differentially affected histone lactylation modification, which was possibly mediated by PFKFB3 driven glycolysis.

Given that the Kla modification can contribute to gene expression^33^, to further elucidate the potential functional significance of H3K18la, we performed CUT&Tag analysis to explore the genome-wide distribution of H3K18la in HUVECs. Analysis of the genome-wide distribution showed the H3K18la binding peaks in CIH, with 14.99% located in promoter regions (≤ 3Kb) (Supplementary Figure 10C). KEGG analysis of H3K18la binding peaks identified 749 candidate genomic loci at metabolic pathways in CIH (Supplementary Figure 10D). The heatmap presented enrichment of H3K18la peaks near transcription start sites (TSSs) with PFKFB3 (Figure 9H). In addition, H3K18la peaks were validated at the promoters of the PFKFB3, which showed an elevated tendency (Figure 9I). Further, a quantitative CUT&Tag-qPCR analysis indicated that the H3K18la levels on PFKFB3 promoters were significantly elevated in HUVECs exposed to CIH, which were reversed by 3PO (Figure 9J). Dual-luciferase reporter assays showed that CIH increased wild-type PFKFB3 promoter activity but not that of a mutant lacking putative H3K18la-responsive elements (Figure 9K). Additionally, inhibition of glycolysis with Oxamate or 2-deoxyglucose (2DG) reduced H3K18la enrichment at the PFKFB3 promoter and decreased PFKFB3 expression (Figures 9K-N). Collectively, these results revealed that H3K18la modification activated the transcription of PFKFB3, indicating a positive feedback loop of PFKFB3-glycolysis-H3K18la that propagated the metabolic rewiring in the context of CIH.

## Discussion

Our study delineated the inflammatory injury of vascular ECs induced by CIH was accompanied by enhanced glycolysis, mitochondrial dysfunction, and impaired pentose phosphate pathway activity, indicating the metabolic-inflammatory crosstalk. We further provided the first evidence that glycolytic reprogramming of ECs under CIH was mainly orchestrated via the glycolytic activator PFKFB3. Genetic or pharmacological inhibition of PFKFB3 could restore the imbalanced glycolytic/mitochondrial metabolism and dampen endothelial inflammation, thereby alleviating vascular injury. Mechanistically, HIF-1α mediated transcriptional upregulation of PFKFB3 and a positive feedback loop of PFKFB3-glycolysis-lactate-H3K18la driving the metabolic-inflammatory regulation were observed for the first time in conditions of CIH. Collectively, these results offer new insights into the critical role of PFKFB3 in mediating glycolytic reprogramming and vascular endothelial injury in response to CIH.

Emerging studies have highlighted the importance of metabolic rewiring in ECs for endothelial activation and atherosclerosis^21^. Here, our transcriptome and *in vitro* analysis of CIH-stimulated ECs revealed upregulation of inflammatory responses, abnormal glycolytic/mitochondrial metabolism, and impaired PPP, indicating the metabolic-inflammatory crosstalk. Furthermore, we identified that PFKFB3 was significantly up-regulated in ECs, rather than other key glycolytic enzymes such as HK2 or LDHA. Subsequent loss- and gain-of-function studies confirmed the critical role of PFKFB3 in mediating the metabolic-inflammatory crosstalk. PFKFB3-driven glycolysis is well documented in endothelial pathophysiology, including abnormal activation, proliferation, migration, vessel sprouting, and phenotype transition^16^, ^18^, ^35^-^37^, and has been implicated in endothelial inflammation triggered by factors like LPS or TNF-α^17^, ^38^-^40^. Our findings establish its critical function within the novel pathophysiological context of CIH/OSA for the first time.

Interestingly, plasma PFKFB3 levels were elevated in both OSA patients and CIH-exposed mice, suggesting its potential as a circulating biomarker. However, the widespread expression of PFKFB3 in multiple tissues (e.g., muscle, adipose) and other vascular cell types (e.g., immune cells, smooth muscle cells) may limit its specificity as a biomarker and complicate interpretation of its origin^41^. In ECs, CIH-induced PFKFB3 upregulation was associated with increased PFKFB3 in the culture medium, which may be released through apoptosis-dependent mechanisms or via extracellular vesicles, though the exact mechanisms await further investigation^14^, ^41^, ^42^. In the present study, inhibition of PFKFB3 using either 3PO or SAC restored abnormal metabolic alternation, suppressed endothelial inflammation, and protected mice from CIH-induced vascular injury.

Indeed, several PFKFB3 inhibitors have been developed that exhibit high specificity for PFKFB3, among which, 3PO is the first and most frequently used in experimental models, demonstrating both efficacy and safety^31^, ^39^, ^40^, ^43^. Using 3PO as a scaffold, other small-molecule inhibitors have been developed and tested in clinical trials with tumor patients^44^. SAC, a phenolic component derived from Salvia miltiorrhiza (Danshen), is clinically prescribed for the treatment of cardiovascular and fibrotic disorders^45^. Previous studies have demonstrated that SAC could reverse PFKFB3 induction by promoting its degradation and therefore limit augmented glycolysis in ECs^18^. Our data showed that either 3PO or SAC could suppress CIH induced increase of blood pressure, inflammatory subsets infiltration, and formation of atherosclerosis, all of which could be reversed by enforced expression of PFKFB3. These results further validate the critical role of PFKFB3, thereby pinpointing a novel therapeutic target for OSA-associated cardiovascular pathology. However, the long-term safety and systemic metabolic consequences of PFKFB3 inhibition remain to be determined. Although preclinical studies have demonstrated acceptable tolerability of PFKFB3 inhibitors^46^, systemic inhibition of glycolysis may affect highly glycolytic tissues such as the retina and immune cells. Tissue-specific or cell-targeting strategies warrant further exploration before clinical translation. Nevertheless, our study provides proof-of-concept evidence that PFKFB3 inhibition is a promising strategy for alleviating CIH-induced vascular injury.

Mechanistic analyses combining bioinformatics, *in vivo* and *in vitro* validation revealed that CIH induced up-regulation of HIF-1α, which bound to the promoter region of PFKFB3 and in turn led to PFKFB3-driven glycolysis and endothelial inflammation. In the context of CVD, hypoxia-inducible factors (HIFs) play the specific and dominant role in the spatiotemporal regulation of oxygen homeostasis^47^. Different from the study that reported the central role of HIF-2α in PFKFB3 mediated expression of growth factors and proinflammatory cytokines in pulmonary hypertension^39^, our results revealed the selective stimulation of HIF-1α (but not HIF-2*α*) induced by CIH to mimic OSA. Differential regulation of HIF-1α and HIF-2*α* by CIH may be partly explained by different hypoxia patterns and durations, which was hypothesized to contribute to oxidative stress and cardiorespiratory pathology^48^.

Lactate, the end-product of glycolysis, is widely known as a metabolic by-product. Recent studies have identified its non-metabolic functions as a precursor which stimulates histone lactylation and directly stimulates gene transcription from chromatin^33^. Histone lactylation serving as an epigenetic modification has been documented in several biological processes and diverse diseases including macrophage polarization^33^, ^49^, ^50^, T cell antitumor immunity^51^, smooth muscle cell proliferation^52^, liver fibrosis^53^, osteoporosis^54^, and tumor progression^55^. In this study, we discovered that the lactate production mediated by the induction of PFKFB3 resulted in an increase in the levels of Pan Kla and H3K18la, indicating the metabolic-epigenetic modification under CIH. Genome-wide analysis further revealed H3K18la enrichment at the PFKFB3 promoter, uncovering the PFKFB3-glycolysis-H3K18la positive feedback loop that exacerbates metabolic rewiring in ECs induced by CIH for the first time.

The underlying mechanisms by which PFKFB3 directly controls the activation of inflammatory responses may include the following possibilities. First, our results revealed upregulated levels of HIF-1α, which is known to play an important role in the regulation of inflammatory responses, including cytokines, chemokines, adhesion protein synthesis and release. Second, several studies have revealed that the effect of PFKFB3 on endothelial inflammation is related to NF-κB signaling. Given that PFKFB3-driven glycolysis is the major source of ATP generation in ECs, it is likely that the activation of NF-κB signaling may be fueled by PFKFB3 expression and activity to support the ATP in response to pro-inflammatory stimulation^40^. Third, given that H3K18la has been reported to regulate the expression of various inflammatory genes, it may also modulate inflammatory mediators in ECs under CIH^56^. Finally, glycolytic byproduct lactate was reported to stimulate inflammatory responses^57^. The data from this study have shown that suppression of PFKFB3 lowered lactate levels in CIH-ECs. Thus, lactate may also be responsible for PFKFB3-mediated activation of ECs. Further studies are warranted to dissect the relative contributions of these intersecting pathways.

Some limitations need to be mentioned here. First, this study focuses on the main pathways of glucose metabolism, other metabolic pathways, including fatty acid oxidation and amino acid metabolism may also play important roles and require further investigation. Second, our study unveils the critical role of PFKFB3 regulating endothelial metabolic reprogramming, but PFKFB3 is also expressed in a variety of tissues and cells, such as smooth muscle cells and macrophages. CIH may induce glycolytic reprogramming in these cells as well, and their crosstalk with ECs, potentially mediated by PFKFB3 itself or its metabolic products, including lactate and inflammatory mediators, may contribute to the overall vascular phenotype in atherosclerosis. Thus, the impact of PFKFB3 on diverse cells and potential cross-talk in the context of atherosclerosis should be further explored. Third, acetylation or phosphorylation of PFKFB3 has been reported to mediate the activation of PFKFB3 in response to multiple external stresses^33^. The epigenetic modification of PFKFB3 and functional consequences should be further explored. Fourth, the H3K18la mechanism remains preliminary. While we have provided functional evidence for H3K18la enrichment at the PFKFB3 promoter, the specific writers, erasers, and readers of this modification in ECs under CIH remain unknown. Moreover, whether H3K18la interacts with other histone modifications including acetylation to regulate gene expression requires further investigation, especially given that acetyl-CoA levels were decreased under CIH. Fifth, our conclusions are largely based on preclinical models; clinical translation will require validation in patient samples and long-term safety assessments of PFKFB3-targeted therapies.

## Conclusions

In conclusion, our study highlights the critical role of PFKFB3 in mediating CIH-induced endothelial glycolytic reprogramming and inflammatory responses. The results also reveal a positive feedback loop of HIF-1α/PFKFB3/H3K18la in the metabolic-inflammatory regulation, offering novel mechanistic insights into the pathogenesis of OSA-related CVD and suggesting potential therapeutic strategies for clinical translation.

## Supplementary Material

Supplementary figures and tables, methods.

## Data Availability

The data are available to academic researchers upon request.

## Abbreviations

2-DG: 2-deoxyglucose
2-ME: 2-methoxyestradiol
3PO: 3-(3-pyridinyl)-1-(4-pyridinyl)-2-propen-1-one
AAV: adeno-associated virus
AHI: apnea hypopnea index
CIH: chronic intermittent hypoxia
CVD: cardiovascular diseases
ECs: endothelial cells
EDN1: endothelin-1
H3K18la: H3K18 lactylation
HIF-1α: hypoxia-inducible factor 1α
HUVECs: human umbilical vein endothelial cells
ICAM-1: intercellular adhesion molecule-1
IL-6: interleukin-6
NC: normoxic control
OSA: obstructive sleep apnea
Pan Kla: pan-lysine lactylation
PFKFB3: phosphofructo-2-kinase/fructose-2,6-biphosphatase 3
PPP: pentose phosphate pathway
SAC: Salvianolic acid C
VCAM-1: vascular cell adhesion molecule-1

## References

[1] Gottlieb DJ, Punjabi NM (2020). Diagnosis and Management of Obstructive Sleep Apnea: A Review. JAMA.

[2] Benjafield AV, Ayas NT, Eastwood PR, Heinzer R, Ip MSM, Morrell MJ (2019). Estimation of the global prevalence and burden of obstructive sleep apnoea: a literature-based analysis. Lancet Respir Med.

[3] Yeghiazarians Y, Jneid H, Tietjens JR, Redline S, Brown DL, El-Sherif N (2021). Obstructive Sleep Apnea and Cardiovascular Disease: A Scientific Statement from the American Heart Association. Circulation.

[4] Javaheri S, Barbe F, Campos-Rodriguez F, Dempsey JA, Khayat R, Javaheri S (2017). Sleep Apnea: Types, Mechanisms, and Clinical Cardiovascular Consequences. J Am Coll Cardiol.

[5] Medina-Leyte DJ, Zepeda-Garcia O, Dominguez-Perez M, Gonzalez-Garrido A, Villarreal-Molina T, Jacobo-Albavera L (2021). Endothelial Dysfunction, Inflammation and Coronary Artery Disease: Potential Biomarkers and Promising Therapeutical Approaches. Int J Mol Sci.

[6] Gimbrone MA Jr, Garcia-Cardena G (2016). Endothelial Cell Dysfunction and the Pathobiology of Atherosclerosis. Circ Res.

[7] Cetin-Atalay R, Meliton AY, Wu D, Woods PS, Sun KA, Peng YJ (2021). Intermittent Hypoxia-Induced Activation of Endothelial Cells Is Mediated via Sympathetic Activation-Dependent Catecholamine Release. Front Physiol.

[8] Emin M, Wang G, Castagna F, Rodriguez-Lopez J, Wahab R, Wang J (2016). Increased internalization of complement inhibitor CD59 may contribute to endothelial inflammation in obstructive sleep apnea. Sci Transl Med.

[9] Gao S, Emin M, Thoma T, Pastellas K, Castagna F, Shah R (2021). Complement promotes endothelial von Willebrand factor and angiopoietin-2 release in obstructive sleep apnea. Sleep.

[10] Zeng X, Guo R, Dong M, Zheng J, Lin H, Lu H (2018). Contribution of TLR4 signaling in intermittent hypoxia-mediated atherosclerosis progression. J Transl Med.

[11] Song D, Fang G, Mao SZ, Ye X, Liu G, Miller EJ (2018). Selective inhibition of endothelial NF-kappaB signaling attenuates chronic intermittent hypoxia-induced atherosclerosis in mice. Atherosclerosis.

[12] Lu Y, Sun Y, Drummer Ct, Nanayakkara GK, Shao Y, Saaoud F (2019). Increased acetylation of H3K14 in the genomic regions that encode trained immunity enzymes in lysophosphatidylcholine-activated human aortic endothelial cells - Novel qualification markers for chronic disease risk factors and conditional DAMPs. Redox Biol.

[13] Peng L, Li Y, Li X, Du Y, Li L, Hu C (2021). Extracellular Vesicles Derived from Intermittent Hypoxia-Treated Red Blood Cells Impair Endothelial Function Through Regulating eNOS Phosphorylation and ET-1 Expression. Cardiovasc Drugs Ther.

[14] Yan YR, Zhang L, Lin YN, Sun XW, Ding YJ, Li N (2021). Chronic intermittent hypoxia-induced mitochondrial dysfunction mediates endothelial injury via the TXNIP/NLRP3/IL-1beta signaling pathway. Free Radic Biol Med.

[15] Eelen G, de Zeeuw P, Simons M, Carmeliet P (2015). Endothelial cell metabolism in normal and diseased vasculature. Circ Res.

[16] De Bock K, Georgiadou M, Schoors S, Kuchnio A, Wong BW, Cantelmo AR (2013). Role of PFKFB3-driven glycolysis in vessel sprouting. Cell.

[17] Schnitzler JG, Hoogeveen RM, Ali L, Prange KHM, Waissi F, van Weeghel M (2020). Atherogenic Lipoprotein(a) Increases Vascular Glycolysis, Thereby Facilitating Inflammation and Leukocyte Extravasation. Circ Res.

[18] Zeng H, Pan T, Zhan M, Hailiwu R, Liu B, Yang H (2022). Suppression of PFKFB3-driven glycolysis restrains endothelial-to-mesenchymal transition and fibrotic response. Signal Transduct Target Ther.

[19] Zhang X, Wang S, Xu H, Yi H, Guan J, Yin S (2021). Metabolomics and microbiome profiling as biomarkers in obstructive sleep apnoea: a comprehensive review. Eur Respir Rev.

[20] Shi L, Pan H, Liu Z, Xie J, Han W (2017). Roles of PFKFB3 in cancer. Signal Transduct Target Ther.

[21] Dzobo KE, Hanford KML, Kroon J (2021). Vascular Metabolism as Driver of Atherosclerosis: Linking Endothelial Metabolism to Inflammation. Immunometabolism.

[22] D'Agostino RB Sr, Vasan RS, Pencina MJ, Wolf PA, Cobain M, Massaro JM (2008). General cardiovascular risk profile for use in primary care: the Framingham Heart Study. Circulation.

[23] Hu D, Yin C, Mohanta SK, Weber C, Habenicht AJ (2016). Preparation of Single Cell Suspensions from Mouse Aorta. Bio Protoc.

[24] Alsaigh T, Evans D, Frankel D, Torkamani A (2022). Decoding the transcriptome of calcified atherosclerotic plaque at single-cell resolution. Commun Biol.

[25] Cao Y, Fu L, Wu J, Peng Q, Nie Q, Zhang J (2022). Integrated analysis of multimodal single-cell data with structural similarity. Nucleic Acids Res.

[26] Long Z, Sun C, Tang M, Wang Y, Ma J, Yu J (2022). Single-cell multiomics analysis reveals regulatory programs in clear cell renal cell carcinoma. Cell Discov.

[27] Harki O, Boete Q, Pepin JL, Arnaud C, Belaidi E, Faury G (2022). Intermittent hypoxia-related alterations in vascular structure and function: a systematic review and meta-analysis of rodent data. Eur Respir J.

[28] Timmerman I, Daniel AE, Kroon J, van Buul JD (2016). Leukocytes Crossing the Endothelium: A Matter of Communication. Int Rev Cell Mol Biol.

[29] Tang XQ, Luo YX, Chen HZ, Liu DP (2014). Mitochondria, endothelial cell function, and vascular diseases. Frontiers in Physiology.

[30] Zhou L, Li J, Wang J, Niu X, Li J, Zhang K (2024). Pathogenic role of PFKFB3 in endothelial inflammatory diseases. Frontiers in Molecular Biosciences.

[31] Clem B, Telang S, Clem A, Yalcin A, Meier J, Simmons A (2008). Small-molecule inhibition of 6-phosphofructo-2-kinase activity suppresses glycolytic flux and tumor growth. Mol Cancer Ther.

[32] Chesney J, Mitchell R, Benigni F, Bacher M, Spiegel L, Al-Abed Y (1999). An inducible gene product for 6-phosphofructo-2-kinase with an AU-rich instability element: Role in tumor cell glycolysis and the Warburg effect. P Natl Acad Sci USA.

[33] Zhang D, Tang ZY, Huang H, Zhou GL, Cui C, Weng YJ (2019). Metabolic regulation of gene expression by histone lactylation. Nature.

[34] Pietrocola F, Galluzzi L, Bravo-San Pedro JM, Madeo F, Kroemer G (2015). Acetyl coenzyme A: a central metabolite and second messenger. Cell Metab.

[35] Feng Y, Zou R, Zhang X, Shen M, Chen X, Wang J (2021). YAP promotes ocular neovascularization by modifying PFKFB3-driven endothelial glycolysis. Angiogenesis.

[36] Perrotta P, Van der Veken B, Van Der Veken P, Pintelon I, Roosens L, Adriaenssens E (2020). Partial Inhibition of Glycolysis Reduces Atherogenesis Independent of Intraplaque Neovascularization in Mice. Arterioscler Thromb Vasc Biol.

[37] Yang K, Qiu T, Zhou J, Gong X, Zhang X, Lan Y (2023). Blockage of glycolysis by targeting PFKFB3 suppresses the development of infantile hemangioma. J Transl Med.

[38] Zhang R, Li R, Liu Y, Li L, Tang Y (2019). The Glycolytic Enzyme PFKFB3 Controls TNF-alpha-Induced Endothelial Proinflammatory Responses. Inflammation.

[39] Cao Y, Zhang X, Wang L, Yang Q, Ma Q, Xu J (2019). PFKFB3-mediated endothelial glycolysis promotes pulmonary hypertension. Proc Natl Acad Sci U S A.

[40] Wang L, Cao Y, Gorshkov B, Zhou Y, Yang Q, Xu J (2019). Ablation of endothelial Pfkfb3 protects mice from acute lung injury in LPS-induced endotoxemia. Pharmacol Res.

[41] Han B, Zhu Z, Wang Y, Zhao N, Chen J, Zhou S (2025). PFKFB3 Promotes Myofibroblast Differentiation and Cardiac Fibrosis Through its Intra- and Extra- Cellular Roles. Journal of Cardiovascular Translational Research.

[42] Chen Q-t, Zhang Z-y, Huang Q-l, Chen H-z, Hong W-b, Lin T (2022). HK1 from hepatic stellate cell-derived extracellular vesicles promotes progression of hepatocellular carcinoma. Nature Metabolism.

[43] Schoors S, De Bock K, Cantelmo AR, Georgiadou M, Ghesquiere B, Cauwenberghs S (2014). Partial and transient reduction of glycolysis by PFKFB3 blockade reduces pathological angiogenesis. Cell Metab.

[44] Wang Y, Qu C, Liu T, Wang C (2020). PFKFB3 inhibitors as potential anticancer agents: Mechanisms of action, current developments, and structure-activity relationships. Eur J Med Chem.

[45] Zhang J, Zhang Q, Liu G, Zhang N (2019). Therapeutic potentials and mechanisms of the Chinese traditional medicine Danshensu. Eur J Pharmacol.

[46] Telang S, O'Neal J, Tapolsky G, Clem B, Kerr A, Imbert-Ferndandez Y (2014). Discovery of a PFKFB3 inhibitor for phase I trial testing that synergizes with the B-Raf inhibitor vemurafenib. Cancer & Metabolism.

[47] Hu YQ, Lu H, Li H, Ge JB (2022). Molecular basis and clinical implications of HIFs in cardiovascular diseases. Trends Mol Med.

[48] Prabhakar NR, Semenza GL (2012). Adaptive and maladaptive cardiorespiratory responses to continuous and intermittent hypoxia mediated by hypoxia-inducible factors 1 and 2. Physiol Rev.

[49] Pan RY, He L, Zhang J, Liu X, Liao Y, Gao J (2022). Positive feedback regulation of microglial glucose metabolism by histone H4 lysine 12 lactylation in Alzheimer's disease. Cell Metab.

[50] Wei L, Yang X, Wang J, Wang Z, Wang Q, Ding Y (2023). H3K18 lactylation of senescent microglia potentiates brain aging and Alzheimer's disease through the NFκB signaling pathway. Journal of Neuroinflammation.

[51] Dan L, Liu L, Sun Y, Song J, Yin Q, Zhang G (2020). The phosphatase PAC1 acts as a T cell suppressor and attenuates host antitumor immunity. Nature Immunology.

[52] Chen J, Zhang M, Liu Y, Zhao S, Wang Y, Wang M (2023). Histone lactylation driven by mROS-mediated glycolytic shift promotes hypoxic pulmonary hypertension. J Mol Cell Biol.

[53] Rho H, Terry AR, Chronis C, Hay N (2023). Hexokinase 2-mediated gene expression via histone lactylation is required for hepatic stellate cell activation and liver fibrosis. Cell Metab.

[54] Wu J, Hu M, Jiang H, Ma J, Xie C, Zhang Z (2023). Endothelial Cell-Derived Lactate Triggers Bone Mesenchymal Stem Cell Histone Lactylation to Attenuate Osteoporosis. Advanced Science.

[55] Xie B, Lin J, Chen X, Zhou X, Zhang Y, Fan M (2023). CircXRN2 suppresses tumor progression driven by histone lactylation through activating the Hippo pathway in human bladder cancer. Molecular Cancer.

[56] Li J, Chen X, Song S, Jiang W, Geng T, Wang T (2025). Hexokinase 2-mediated metabolic stress and inflammation burden of liver macrophages via histone lactylation in MASLD. Cell Reports.

[57] Végran F, Boidot R, Michiels C, Sonveaux P, Feron O (2011). Lactate Influx through the Endothelial Cell Monocarboxylate Transporter MCT1 Supports an NF-κB/IL-8 Pathway that Drives Tumor Angiogenesis. Cancer Research.

