# Supplementary material for: PFKFB3 Mediated Glycolytic Reprogramming Drives Vascular Endothelial Injury Under Chronic Intermittent Hypoxia
Source: Int J Biol Sci. 2026 May 22;22(11):5735–53. doi: 10.7150/ijbs.129280 (PMC13282744; doi:10.7150/ijbs.129280)
Supplement: Supplementary file 1 — Supplementary figures and tables, methods. [file ijbsv22p5735s1.pdf]

PFKFB3 Mediated Glycolytic Reprogramming Drives Vascular Endothelial Injury Under Chronic Intermittent Hypoxia

SUPPLEMENTAL INFORMATION

Supplementary figures

Supplementary Figure. 1-10

Supplementary tables

Supplementary Table.1 Demographic characteristics of the subjects.

Supplementary Table.2 Primer sequences for RT-qPCR.

Supplementary Table.3 Key resources table.

Method details

Supplementary figures and figure legends

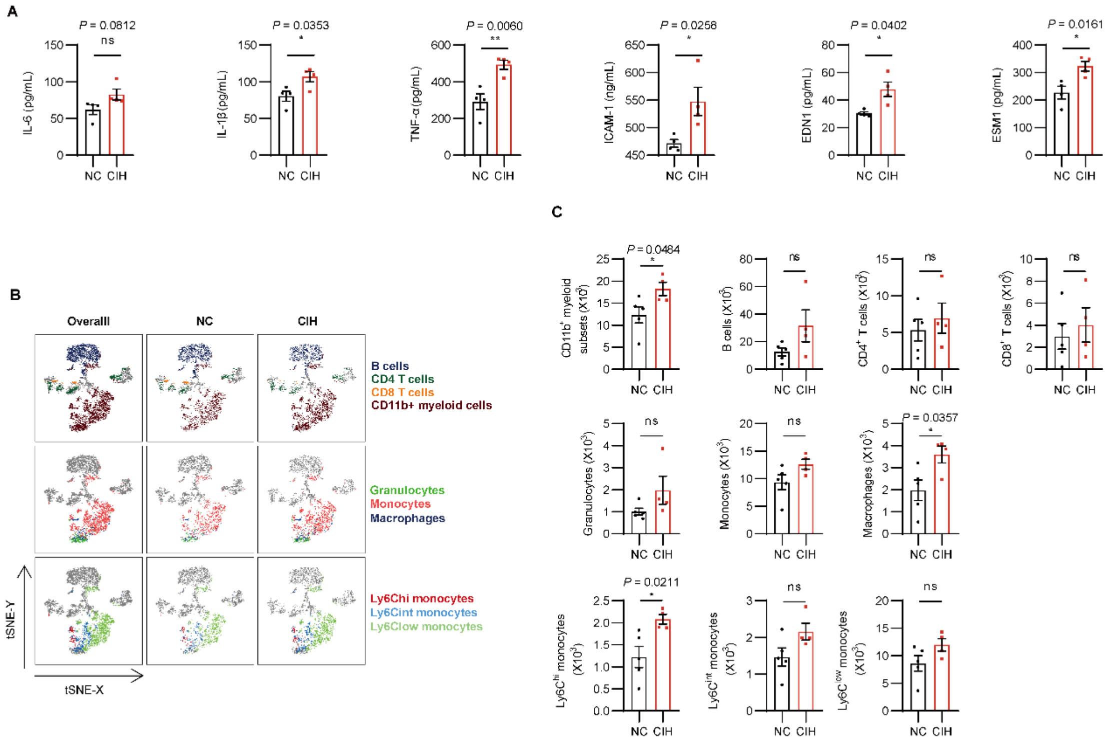

Supplementary Figure 1. CIH exacerbates vascular injury in APOE$^{-/-}$ mice. (related to Figure 1). (A) Systemic serum cytokines (IL-6, IL-1β, TNF-α, ICAM-1, EDN1, and ESM1) in APOE$^{-/-}$ mice subjected to NC or CIH for 8 weeks (n = 4 per group). **(B)** T-distributed stochastic neighbor embedding (t-SNE) visualization of CD45^{+} live cells from aortic single-cell suspensions. Cell populations defined by the manual gating strategy were projected onto t-SNE maps and assigned specific colors. **(C)** Quantification using flow cytometry of CD11b^{+} myeloid cells, B cells, CD4^{+} T cells, CD8^{+} T cells, granulocytes, monocytes, macrophages, Ly6C^{hi}, Ly6C^{int}, and Ly6C^{low} monocytes in the aortas of APOE$^{-/-}$ mice after 8 weeks of NC or CIH (n = 4-5 per group). The data are presented as mean ± SEM. Statistical significance was determined by unpaired Student's t-test. Ns, no statistical significance, *P < 0.05, **P < 0.01, ***P < 0.001.

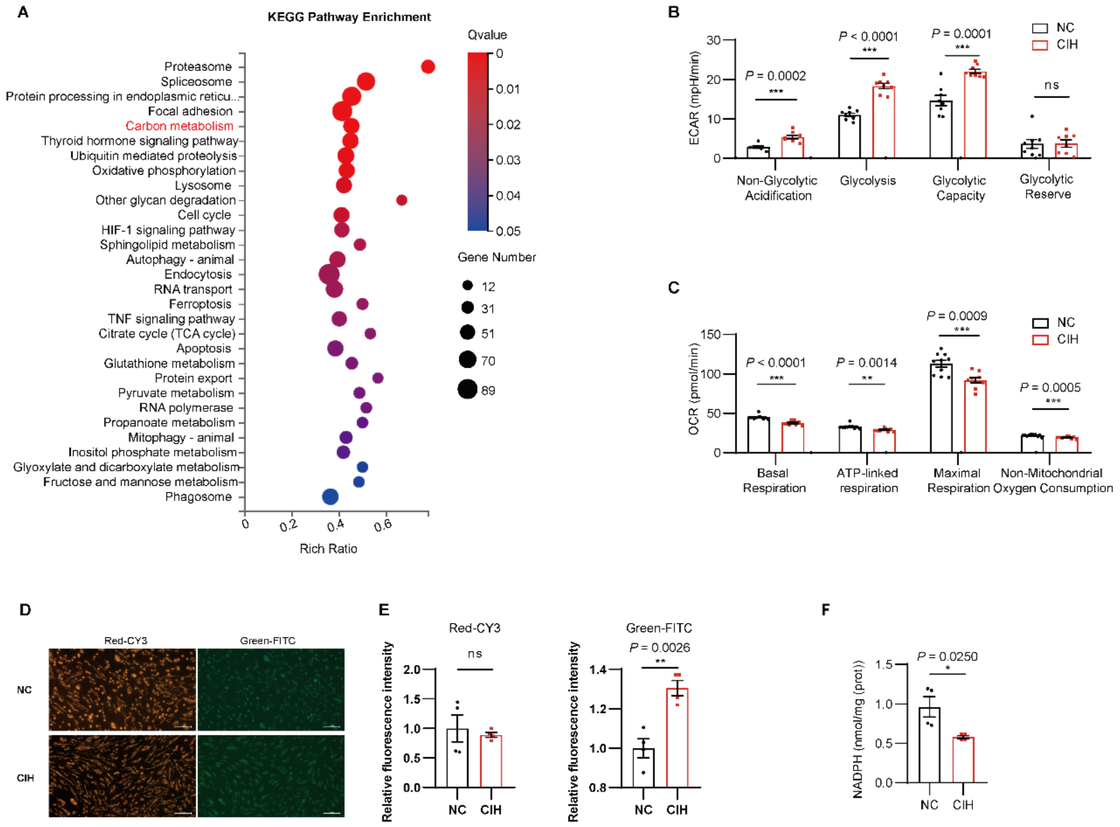

Supplementary Figure 2. Rewiring of glucose metabolism under CIH (related to Figure 2). (A) KEGG enrichment analysis of differentially expressed genes between NC and CIH groups. **(B)** Glycolytic flux measured by Seahorse Flux Analysis of HUVECs treated with NC or CIH by recording extracellular acidification rate (ECAR) after injection of glucose, oligomycin, and 2deoxyglucose (2DG). Glycolysis, Glycolytic capacity, Glycolytic reserve, and non-glycolytic acidification were obtained (n = 8 per group). **(C)** Mitochondrial respiration assessed by oxygen consumption rate (OCR) after injection of oligomycin, carbonyl cyanide-4-(trifluoromethoxy)phenylhydrazone (FCCP), antimycin A and rotenone. Basal respiration, ATP-linked respiration, maximal respiration, and non-mitochondrial oxygen consumption were obtained (n = 10 per group). **(D)** Mitochondrial membrane

potential was detected by JC-1 at 24 h after NC or CIH treatment. Scale bar, 25 μm. **(E)** Quantification of relative fluorescence intensity in (D) (n = 4 per group). **(F)** Cellular NADPH abundance between NC and CIH group (n = 4 per group). The data were presented as mean ± SEM. Statistical significance was determined by unpaired Student's t-test. Ns, no statistical significance, **P* < 0.05, ***P* < 0.01, ****P* < 0.001.

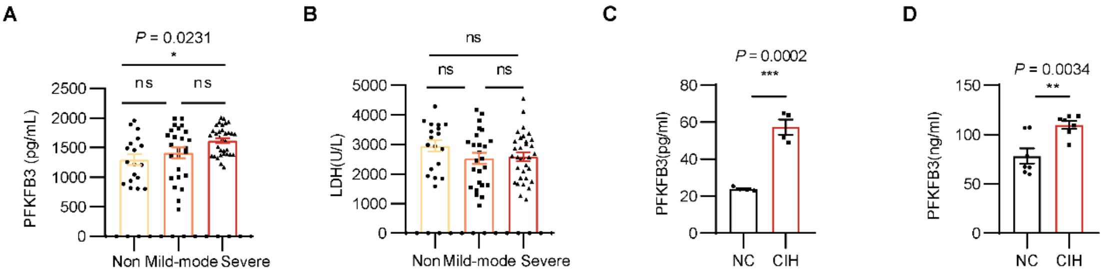

Supplementary Figure 3. CIH upregulates PFKFB3 expression (related to Figure 3). (A, B) The levels of plasma PFKFB3 and LDH among non-obstructive sleep apnea (OSA), mild-moderate OSA, and severe OSA groups. **(C)** Levels of PFKFB3 in culture medium of HUVECs under NC or CIH (n = 4 per group). **(D)** Plasma PFKFB3 levels in C57BL/6J mice after 8 weeks of NC or CIH (n = 6 per group). The data are presented as mean ± SEM. Statistical significance was determined by unpaired two-tailed Student's t-test (C, D), or one-way ANOVA followed by post hoc test (A, B). Ns, not significant; **P* < 0.05, ***P* < 0.01, ****P* < 0.001.

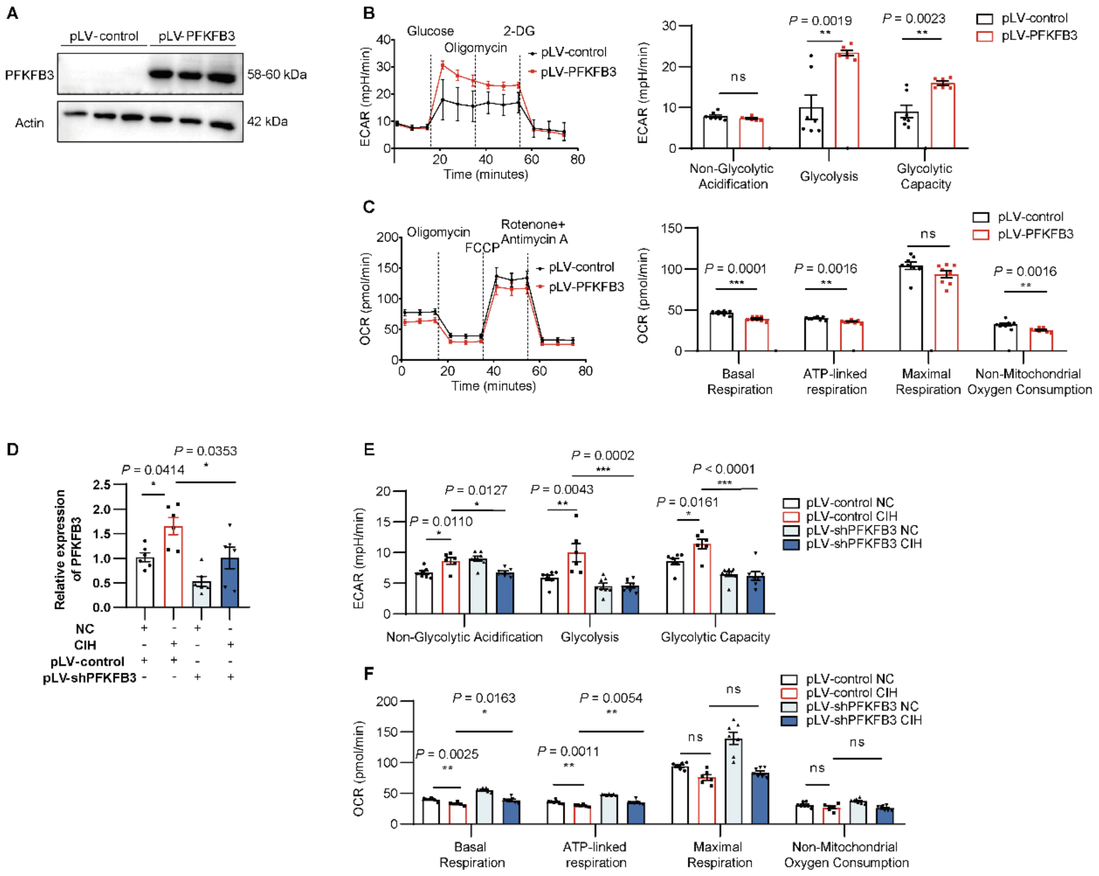

Supplementary Figure 4. PFKFB3 regulates endothelial metabolism reprogramming (related to Figure 4). (A) Western blots of PFKFB3 in HUVECs transfected with either pLV-PFKFB3 or pLV-control. **(B, C)** Glycolytic flux (ECAR) and mitochondrial respiration (OCR) in HUVECs transfected with either pLV-PFKFB3 or pLV-control (n = 6-8 per group). **(D)** Relative mRNA levels of PFKFB3 in HUVECs transfected with either pLV-shPFKFB3 or pLV-control followed by NC or CIH (n = 6 per group). **(E, F)** Glycolytic flux (ECAR) and mitochondrial respiration (OCR) in HUVECs transfected with either pLV-shPFKFB3 or pLV-control followed by NC or CIH (n = 6-8 per group). The data are presented as mean ± SEM. Statistical significance was determined by unpaired Student's t-test (**B, C**) or ANOVA (**E, F**). Ns, no statistical significance, *$P < 0.05$, **$P < 0.01$, ***$P < 0.001$.

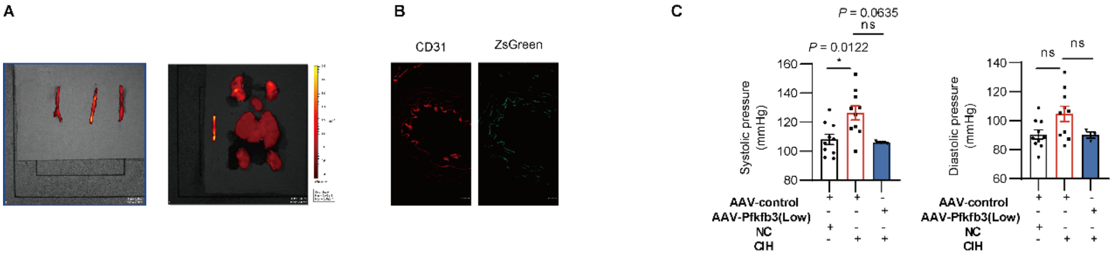

Supplementary Figure 5. PFKFB3 mediates vascular injury induced by CIH (related to Figure 4). (A) Representative bioluminescence images of aortas from untreated, HBAAV2/VEC-TIE-ZsGreen-injected, or PBS-injected, or mice (left). Bioluminescence imaging of aorta, lung, heart, liver, and kidney from mice injected with HBAAV2/VEC-TIE-ZsGreen. (right). **(B)** Immunofluorescence of aortic sections from AAV-ZsGreen-injected mice, co-stained for CD31 (red). Scale bar, 20 μm. **(C)** Systolic and diastolic blood pressure in C57BL/6J mice transfected with AAV-control or AAV-Pfkfb3(Low) and followed by NC or CIH for 8 weeks(n = 3-10 per group). The data are presented as mean ± SEM. Statistical significance was determined by ANOVA. Ns, no statistical significance, **P* < 0.05, ***P* < 0.01, ****P* < 0.001.

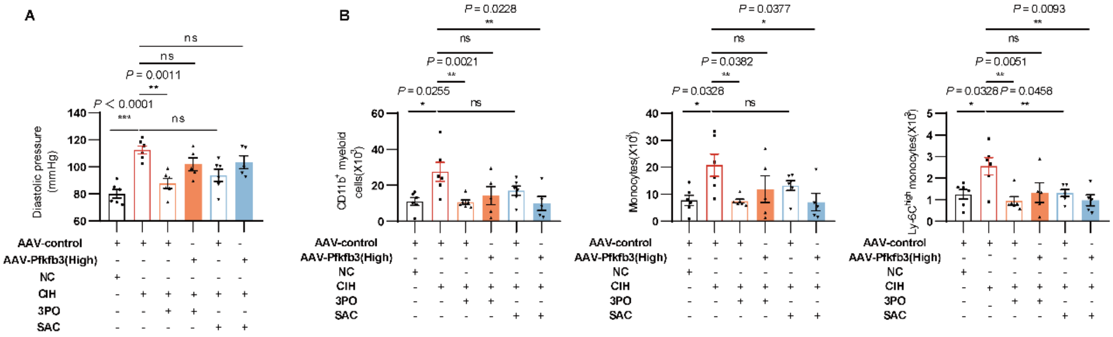

Supplementary Figure 6. PFKFB3 inhibition alleviates CIH-induced vascular injury. (related to Figure 5). APOE$^{-/-}$ mice were grouped by AAV-control-NC, AAV-control-CIH, AAV-control-CIH+3PO, AAV-Pfkfb3(high)-CIH+3PO, AAV-control-CIH+SAC, or AAV-Pfkfb3(high)-CIH+SAC. **(A)** Diastolic blood pressure in each

group (n = 5-7 per group). **(B)** Quantification using flow cytometry of CD11b^{+} myeloid cells, monocytes, and Ly6C^{hi} monocytes in aortic single-cell suspensions (n = 5-7 per group). The data are presented as mean ± SEM. Statistical significance was determined by ANOVA. Ns, no statistical significance, **P* < 0.05, ***P* < 0.01, ****P* < 0.001.

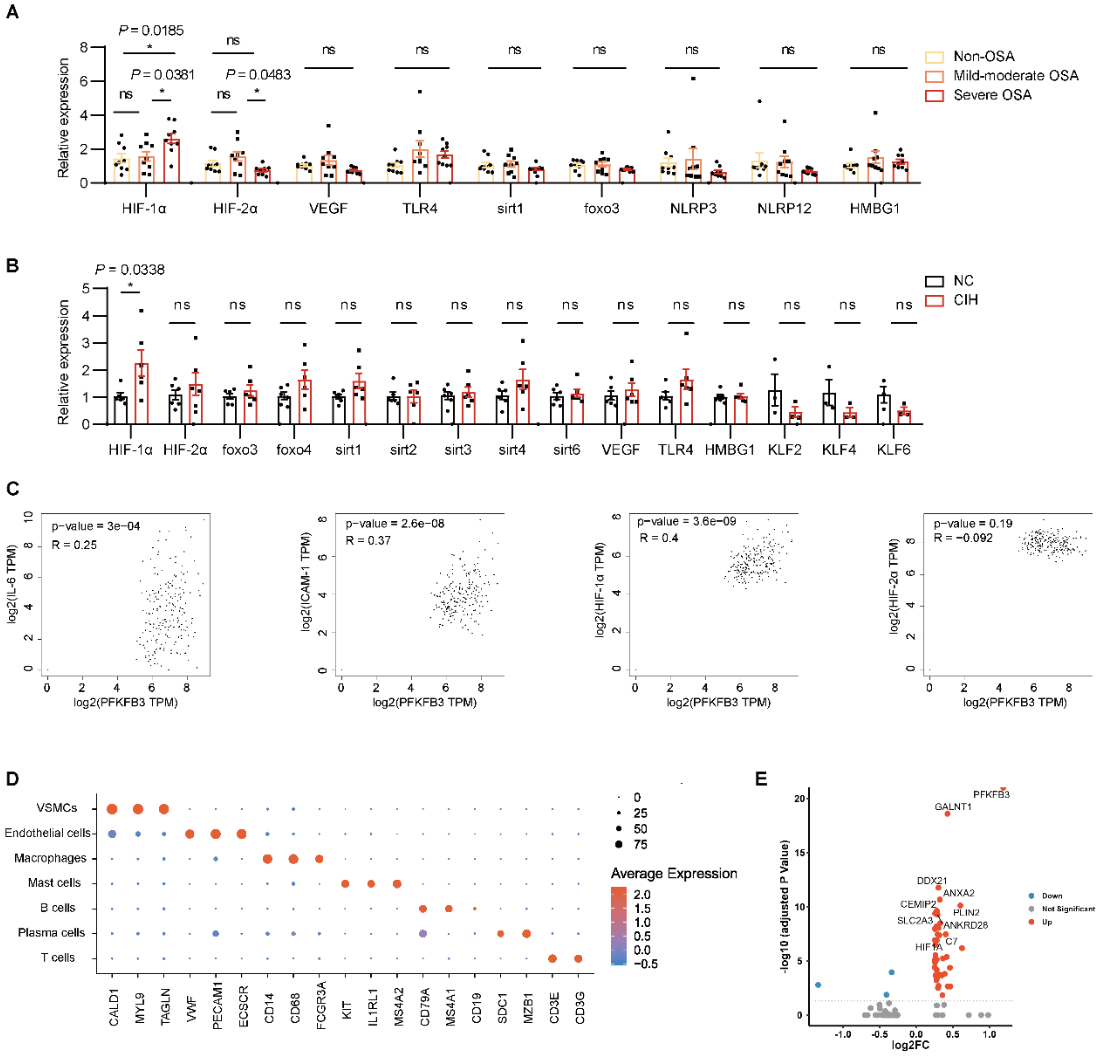

Supplementary Figure 7. CIH activates HIF-1α and correlation analysis between PFKFB3 and HIF-1α (related to Figure 7). (A) Relative mRNA levels of HIF-1α, HIF-2α, VEGFA, TLR4, SIRT1, FOXO3, NLRP3, NLRP12, and HMBG1in HUVECs

treated with 20% plasma from non-OSA, mild to moderate OSA, or severe OSA (n = 9-10 per group). **(B)** Relative mRNA levels of HIF-1α, HIF-2α, VEGF, sirt1, sirt 2, sirt 3, sirt 4, sirt 6, foxo3, foxo4, TLR4, HMBG1, KLF2, KLF4, and KLF6 in HUVECs under NC or CIH (n = 3-6 per group). **(C)** Correlation analysis between PFKFB3 and IL-6, ICAM-1, HIF-1α, or EPAS1 (HIF-2α) in in human aortas using GTEx database via GEPIA. **(D)** Dot-plot depicting cell-type marker genes, resulting in the identification of vascular smooth muscle cells, endothelial cells, macrophages, mast cells, B cells, plasma cells, and T cells from scRNA-seq analysis based on carotid artery tissue. **(E)** Volcano plot of differentially expressed genes in PFKFB3-high ECs versus PFKFB3-low ECs from scRNA-seq analysis based on carotid artery tissue. Red and blue indicate up- and down-regulated genes, respectively (adjusted $P \leq 0.05$). The data were presented as mean ± SEM. Statistical significance was determined by ANOVA (**A**) or unpaired Student's test (**B**). Ns, no statistical significance, $*P < 0.05$, $**P < 0.01$, $***P < 0.001$.

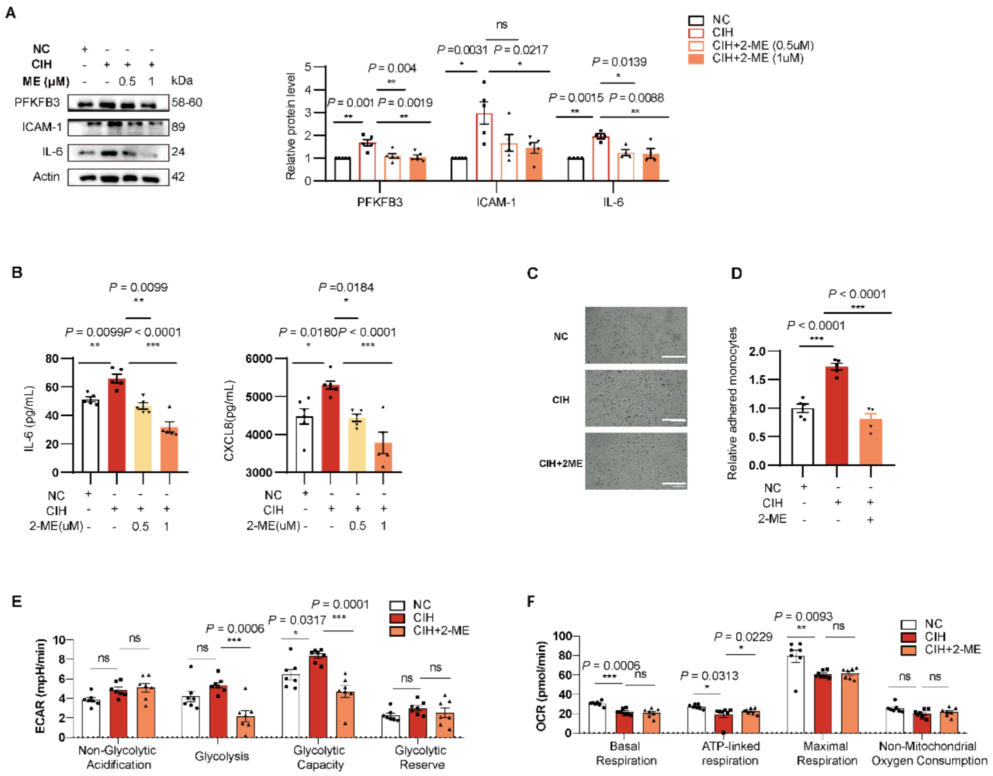

Supplementary Figure 8. HIF-1α regulates PFKFB3 driven metabolic reprogramming and endothelial inflammation (related to Figure 7). (A) Western blots of PFKFB3, ICAM-1, and IL-6 in HUVECs treated with NC, CIH, or CIH + 2-ME (n = 4-5 per group). **(B)** Levels of IL-6 and CXCL8 in cell culture medium (n = 5 per group). **(C)** Representative images of monocytes adhered to HUVECs. Adhered monocytes are visualized as dark round cells. Scale bar, 20 μm. **(D)** Quantification of adherent monocytes in (C)(n = 5 per group). **(E, F)** Glycolytic flux (ECAR) and mitochondrial respiration (OCR) in HUVECs in each group (n = 7 per group). The data are presented as mean ± SEM. Statistical significance was determined by ANOVA. Ns, no statistical significance, *$P < 0.05$, **$P < 0.01$, ***$P < 0.001$.

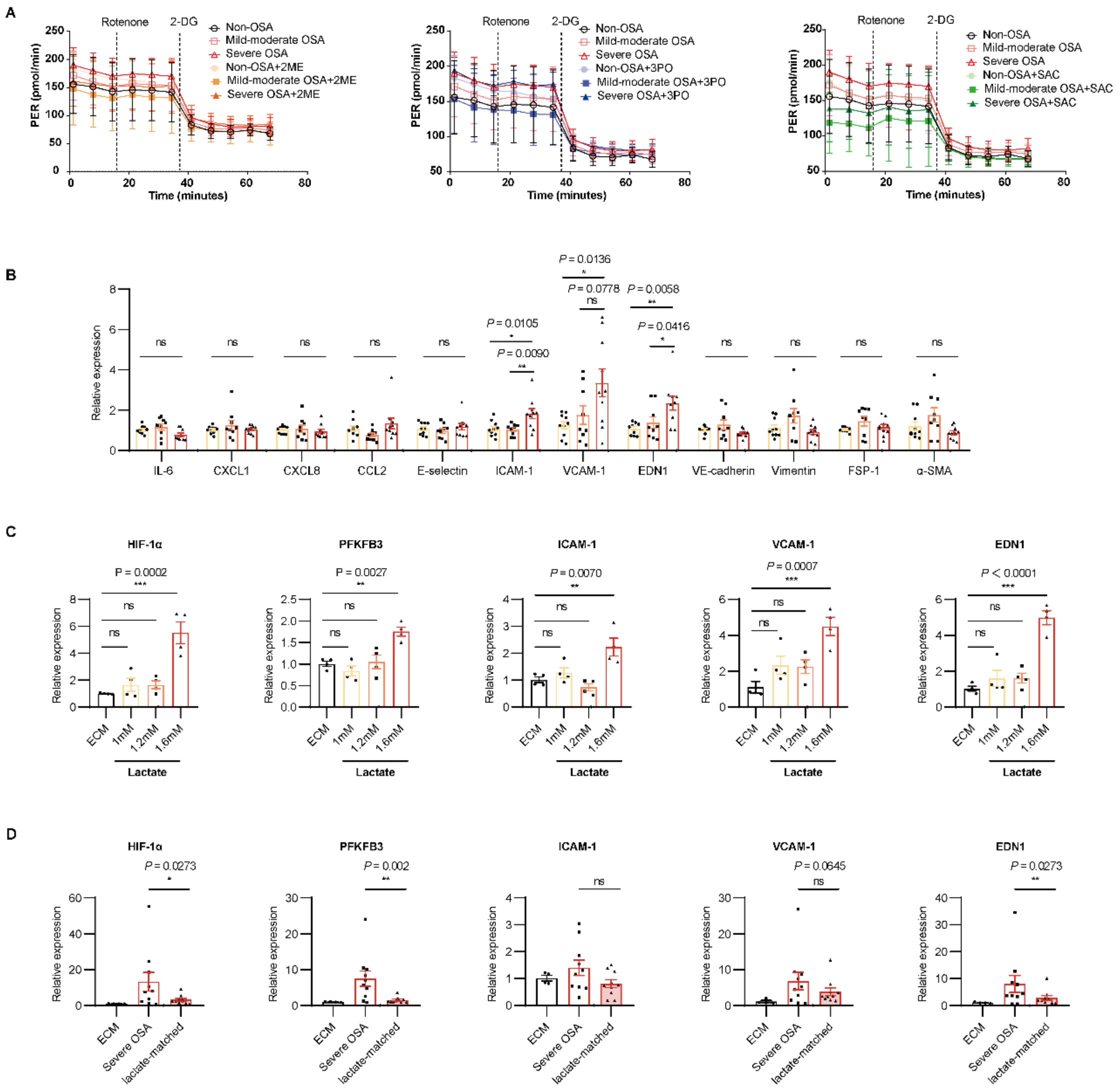

Supplementary Figure 9. Targeting the HIF-1α-PFKFB3 axis alleviates OSA induced endothelial inflammation and glycolysis. (related to Figure 8). (A) Glycolytic rate assay of HUVECs treated with plasma alone or with 2-ME, 3PO, or SAC (n = 5-6 per group). **(B)** Relative mRNA expression levels of cytokines (IL-6), chemokines (CXCL1, CXCL8, and CCL2), adhesion molecules (E-selectin, ICAM-1, and VCAM-1), EC specific molecules (EDN1, and VE-cadherin) and EndMT related molecules (vimentin, FSP-1, and α-SMA) in HUVECs treated with 20% plasma from non-OSA, mild to moderate OSA, and severe OSA (n = 9-10 per group). **(C)** Relative mRNA expression levels of HIF-1α, PFKFB3, ICAM-1, VCAM-1, and EDN1 in

HUVECs treated with ECM or the lactate at the average concentrations of non-OSA, mild-moderate OSA, or severe OSA group (n = 4 per group). **(D)** Relative mRNA expression levels of HIF-1α, PFKFB3, ICAM-1, VCAM-1, and EDN1 in HUVECs treated with 20% severe OSA plasma or lactate at the matched concentration (n = 10 per group). The data were presented as mean ± SEM. Statistical significance was determined by ANOVA (B-C) or paired t-test (D). Ns, no statistical significance, **P* < 0.05, ** *P* < 0.01, *** *P* < 0.001.

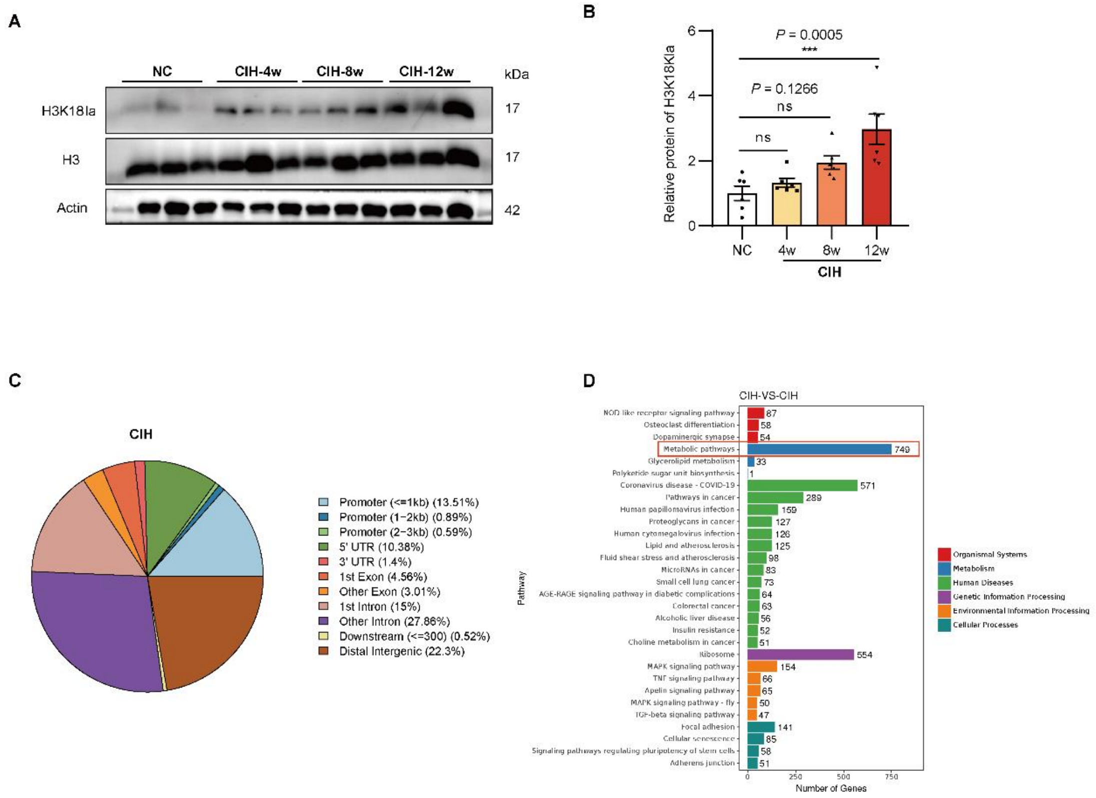

Supplementary Figure 10. PFKFB3–lactate–H3K18la positive feedback in CIH (related to Figure 9). (A) Representative Western blots of H3K18la in aortas from C57BL/6J mice of NC, CIH-4w, CIH-8w, or CIH-12w. **(B)** Quantification of (A) (n = 6 per group). **(C)** Genome-wide distribution of up-regulated H3K18la-binding peaks

in HUVECs treated with CIH. **(D)** KEGG analysis of the H3K18la binding peaks in HUVECs treated with CIH. The data were presented as mean ± SEM. Statistical significance was determined by ANOVA. Ns, no statistical significance, $^{*}P < 0.05$, $^{**}P < 0.01$, $^{***}P < 0.001$.

Supplementary Table.1 Demographic characteristics of the subjects.

	Non-OSA	Mild-moderate OSA	Severe OSA	P value
	n = 18	n = 25	n = 33	
Age, years	36(28.5-48.5)	43.5(35.25-50)	43(36.25-53.75)	0.058
Male gender, n (%)	15(83.3%)	21(84%)	32(97%)	0.147
BMI, Kg/m^2	24.76±3.82	26.12±3.22	28.89±4.31	0.001**
NC, cm	38.94±4.07	40.26±3.18	41.96±3.31	0.012*
WC, cm	98.53±11.98	96.54±10.95	97.77±14.23	0.881
Smoking, n (%)	15(83.3%)	20(83.3%)	21(67.7%)	0.339
Hypertension, n (%)	13(72.2%)	14(58.3%)	13(41.9%)	0.111
PSQI	8.28±3.2	7.42±2.9	7.94±3.35	0.692
ESS	10.78±4.82	9.83±4.29	12.42±5.52	0.157
AHI, events/h	1.67(1.31-2.78)	19.28(12.68-27.34)	69.79(44.26-78.92)	0.000***
$LSpO_2$ (%)	92(89.5-93)	85(79-87.75)	65(57-76)	0.000***
Mean SpO_2 (%)	96(95.5-97)	95(95-96)	91(90-94)	0.000***

TS90, min	0.1(0-0.1)	2.05(0.3-14.48)	132(26.78-202.58)	0.000***
FBG, mmol/L	4.2(3.98-5.06)	4.75(4.29-5.81)	4.73(4.29-5.64)	0.053
Pre-sleep SBP, mmHg	141.06±15.99	138.26±14.97	143.3±20.45	0.595
Pre-sleep DBP, mmHg	89.06±11.87	85.39±11.15	88.9±14.48	0.554
Morning SBP, mmHg	138.88±20.74	138.43±14.54	137.5±18.85	0.963
Morning DBP, mmHg	86.09±11.09	86.88±13.76	87.29±13.14	0.676
TC, mmol/L	4.14(3.82-4.81)	4.9(4.39-5.09)	4.13(3.75-4.63)	0.06
TG, mmol/L	1.69(1.27-2.69)	2.3(1.35-2.85)	2.25(1.66-3.06)	0.091
HDL-C, mmol/L	1.15(0.91-1.29)	1.32(1.18-1.5)	1.23(0.87-1.5)	0.333
LDL-C, mmol/L	2.47(1.73-2.92)	2.81(2.49-3.1)	2.63(2.2-3.12)	0.026*
Uric acid, μmol/L	465.67(388.06-605.97)	420.9(349.25-565.67)	510.45(341.79-626.87)	0.470
Framingham CVD risk, %	4.32(1.74-11.61)	5.88(4.73-12.9)	12.08(3.78-21.35)	0.016*

The data were shown as n (%), mean ± SD, or median (interquartile range, 25–75%). Statistical significance was determined by ANOVA or the Kruskal–Wallis test.

Abbreviations: OSA, obstructive sleep apnea; BMI, body mass index; NC, neck circumference; WC, waist circumference; PSQI, Pittsburgh Sleep Quality Index; ESS, Epworth Sleepiness Scale; AHI, apnea hypopnea index; $LSpO_2$, lowest oxygen saturation; TS90, time spent below oxygen saturation of 90%; FBG, fast blood glucose; SBP, systolic blood pressure; DBP, diastolic blood pressure; TC, total cholesterol; TG, triglycerides; HDL-C, high-density lipoprotein cholesterol; LDL-C, low-density lipoprotein cholesterol.

Supplementary Table.2 Primer sequences for RT-qPCR.

Gene	Gene ID	Forward primer	Reverse primer
β-actin	60	ATCATGTTTGAGACCTTCAACA	CATCTCTTGCTCGAAGTCCA
IL-6	3569	AGTGCCTCT TTGCTGCTTTCAC	TGACAAACAAATTCGGTACATCCT
IL-1β	3553	ATGATGGCTTATTACAGTGGCAA	GTCGGAGATTCGTAGCTGGA
TNF-α	7124	CTCTTCTGCCTGCTGCACTTTG	ATGGGCTACAGGCTTGTCACTC
CXCL1	2919	AGCTTGCCTCAATCCTGCATCC	TCCTTCAGGAACAGCCACCAGT
CXCL8	3576	GAGAGTGATTGAGAGTGGACCAC	CACAACCCTCTGCACCCAGTTT
CCL2	6347	GCTCATAGCCACCTTCATTC	GGACACTTGCTGCTGGTGATTC
E-Selectin	6401	GCACTGTGTGCAAGTTCGC	GGCTTTTGGTAGCTTCCGTC
ICAM-1	3383	GCTTCGTGTCCTGTATGGC	CTGGCGTTATAGAGGTACG
VCAM-1	7412	TTCTGAGAGTGTCAAAGAAGG	AAGGAGGATGCAAAATAGAGC
EDN1	1906	AAGGCAACAGACCGTGAAAAT	CGACCTGGTTTGTCTTAGGTG
ESM1	11082	GGCGGCACCACCATGTACCCT	GCCATGTCATGCTCTTTGCAG
VE-cadherin	1003	TTGGAACCAGATGCACATTGAT	TCTTGCGACTCACGCTTGAC
α-SMA	59	AAAAGACAGCTACGTGGGTGA	GCCATGTTCTATCGGGTACTTC
FSP-1	7431	AAATGGCTCGTCACCTTCG	AGAAATCCTGCTCTCCTCGC
S100A4	6275	GATGAGCAACTTGGACAGCAA	CTGGGCTGCTTATCTGGGAAG
GLUT1	6513	CTTCCAGTATGTGGAGCAACTGT	GCACAGTGAAGATGATGAAGACG
HK2	3099	TTGACCAGGAGATTGACATGGG	CAACCGCATCAGGACCTCA
PFKM	5213	TTGGGGGCTTTGAGGCTTAC	GAGCCAGGGACATTGTTGGA
PKM	5315	ATTATTTGAGGAACTCCGCCG	ATTCCGGGTCACAGCAATGAT

LDHA	3939	ATGGAGATTCCAGTGTGCCTGT	CAGAGAGACACCAGCAACATTC
PFKFB1	5207	GGCGAGAGTGAACTCAACATCAG	TGTGACTGGTCCACACCTTCAG
PFKFB2	5208	ACTTAACGTGGAGGCTGTGAA	TCATCCTTACAGGGGTTTGG
PFKFB3	5209	ATTGCGGTTTTCGATGCCAC	GCCACAACTGTAGGGTCGT
PFKFB4	5210	GATCCTGAGGTCATAGCTGCCA	CTATCCAGGTCCTCATCTAGCG
HIF-1α	3091	GAAACTTCTGGATGCTGGTGATTT	GCAATTCATCTGTGCTTTCATGTCA
HIF-2α	2034	CTGTGTCTGAGAAGAGTAACTTCC	TTGCCATAGGCTGAGGACTCCT

The primer sequences for CUT&Tag-qPCR

Gene	Forward primer	Reverse Primer
PFKFB3-1	CTGTCTGGGTGTCGCGG	GTCACCGGGACTCCTACCT
PFKFB3-2	AGGCTTTGACCAAATATGTCCCT	GACACCCAGACAGGACGAC
Spike	GCCTTCTTCCCATTTCTGATCC	CACGAATCAGCGGTAAAGGT

Supplementary Table.3 Key resources table.

REAGENT or RESOURCE	SOURCE	IDENTIFIER
Antibodies		
Anti-PFKFB3	abcam	ab181861
Human/Mouse/Rat CD31/PECAM-1 Antibody	R&D Systems	AF3628
Anti-IL-6 Rabbit pAb	Servicebio	GB11117-100
Anti-ICAM-1 Rabbit pAb	Servicebio	GB11106-100
Acetyl-Histone H3 (Lys9) (C5B11) Rabbit mAb	Cell Signaling Technology	9649T
Acetyl-Histone H3 (Lys14) (D4B9) Rabbit mAb	Cell Signaling Technology	7627T
Acetyl-Histone H3 (Lys27) (D5E4) XP® Rabbit mAb	Cell Signaling Technology	8173T
Histone H3 (D1H2) XP Rabbit mAb 4499	Cell Signaling Technology	4499T
Anti-L-Lactyl Lysine Rabbit mAb	PTM BIO	PTM-1401RM
Anti-L-Lactyl-Histone H3 (Lys18) Rabbit mAb-ChIP Grade	PTM BIO	PTM-1427RM
Anti-L-Lactyl-Histone H3 (Lys14) Rabbit mAb	PTM BIO	PTM-1414RM
Anti-L-Lactyl-Histone H3 (Lys9) Rabbit mAb	PTM BIO	PTM-

		1419RM
Anti-L-Lactyl-Histone H3 (Lys56) Rabbit mAb	PTM BIO	PTM-1421RM
Fixable Viability Stain 620 100ug	BD Pharmingen	564996
Ms CD45 PerCP-Cy5.5 30-F11 100ug	BD Pharmingen	550994
CD11b BV510 M1/70 50ug	BD Pharmingen	562950
Ms Ly-6C BV605 AL-21 50ug	BD Pharmingen	563011
Ms Ly-6G PE-Cy7 1A8 50ug	BD Pharmingen	560601
Ms CD45R/B220 BV786 RA3-6B2 50ug	BD Pharmingen	563894
Ms CD4 FITC RM4-5 100ug	BD Pharmingen	553046
Ms CD8a APC-Cy7 53-6.7 100ug	BD Pharmingen	557654
BD Pharmingen™ APC Hamster Anti-Mouse CD11c	BD Pharmingen	550261
Ms CD3e PE-Cy5 145-2C11 100ug	BD Pharmingen	553065
BD Pharmingen™ PE Rat Anti-Mouse F4/80	BD Pharmingen	565410
BV421 Rat Anti-Mouse CD86(GL1)	BD Pharmingen	564198
BUV496 Rat Anti-Mouse CD86	BD Pharmingen	750437
Anti -β-actin	Servicebio	GB15001-100
Anti -IL-6	Aifang biological	AF06790
Anti -E-selectin	Beyotime	AF7959
Anti -ICAM-1	Beyotime	AF1774
Anti -EDN1	Proteintech	12191-1-AP

Anti -HK2	Santa Cruz	sc-130358
Anti -LDHA	Santa Cruz	sc-133123
Anti -HIF-1α	Santa Cruz	sc-13515
Anti -HIF-2α	Santa Cruz	sc-13596
Chemicals, Enzymes, Reagents and recombinant proteins		
HFD（60 Kcal% Fat High Fat Feed）	Jiangsu Xietong Pharmaceutical Bio-engineering Co., Ltd	XTHF60
3PO	MCE	HY-19824
3PO	Targetmo	T3260
SAC(Salvianolic Acid C)	Targetmol	T3149
2-ME (2-Methoxyestradiol)	MCE	HY-12033
Oxamate	MCE	HY-W013032A
2DG	Agilent	N/A
2-NBDG	thermofisher	N13195
CELLROX GREEN REAGENT	ThermoFisher	C10444
Collagenase I	Yeasen	40507ES60
Collagenase II	Yeasen	40508ES60
DNase I	Yeasen	10608ES25
Hyaluronidase	Yeasen	20426ES60
CD31 MicroBeads	Miltenyi Biotec	130-097-418

AAV-Pfkfb3(High)	Hanbio	79050235
AAV-Pfkfb3(Low)	Hanbio	79050237
pLV[Exp]-EGFP:T2A:Puro-EF1A>FLAG/hPFKFB3[NM_001363545.2] (pLV-PFKFB3)	VectorBuilder	N/A
pLV[shRNA]-EGFP:T2A:Puro-U6>hPFKFB3 (pLV-shPFKFB3)	VectorBuilder	N/A
HIF-1α siRNA	Aisen	N/A
Seahorse XFe96 FluxPak mini.	Agilent	102601-100
Seahorse XFp Media & Calibrant	Agilent	103575-100
Seahorse XF 1.0 M glucose solution, 50 mL	Agilent	103577-100
Seahorse XF 100 mM pyruvate solution, 50 mL	Agilent	103578-100
Dual-Luciferase Reporter Assay System	Yeason	11402ES60
Critical Commercial Assays		
Total cholesterol assay kit	Nanjing Jiancheng Bioengineering Institute	A111-1-1
Triglyceride assay kit	Nanjing Jiancheng Bioengineering Institute	A110-1-1
High-density lipoprotein cholesterol assay kit	Nanjing Jiancheng Bioengineering	A112-1-1

	Institute	
Low-density lipoprotein cholesterol assay kit	Nanjing Jiancheng Bioengineering Institute	A113-1-1
Glucose kit (glucose oxidase method)	Nanjing Jiancheng Bioengineering Institute	A154-1-1
Uric acid (UA) Test Kit	Nanjing Jiancheng Bioengineering Institute	C012-2-1
RayPlex®Human Inflammation Array Kit 1	RayBiotech	FAH-INF-1-96
Human 6-phosphofructokinase2 (PFKFB3) ELISA Kit	Aifang biological	AF11277-A
6-Plex Mouse ProcartaPlex Panel	LAIZEE BIOTECH Co., LTD	PPX-06
Mouse Endothelin-1 ELISA Kit	Invitrogen(thermofisher)	EM26RB
Endocan (ESM1) Mouse ELISA Kit	Invitrogen(thermofisher)	EMESM1
Mouse ICAM-1/CD54(intercellular adhesion molecule 1) ELISA Kit	Elabscience	E-EL-M3037

Mouse ET-1(Endothelin 1) ELISA Kit	Elabscience	E-EL-M2730
Mouse Interleukin 6 (IL-6) ELISA Kit	Aifang biological	N/A
Mouse Interleukin 1β (IL-1β) ELISA Kit	Aifang biological	N/A
Mouse Tumor necrosis factor α (TNF-α) ELISA Kit.	Aifang biological	N/A
Mouse Endocan; ESM-1 (Endocan; ESM-1) ELISA Kit	Aifang biological	N/A
Mouse 6-phosphofructokinase2 (PFKFB3) ELISA Kit	Aifang biological	AF43378-A
Mouse MCP-1/CCL2 ELISA Kit	Dayou	1217392
Human IL-6 ELISA Kit	Beyotime	PI330
Human IL-1β ELISA Kit	Beyotime	PI305
Human ICAM-1 ELISA Kit	Proteintech	KE00061-96T
Human IL-8 ELISA Kit	Dayou	1110802
Human MCP-1 ELISA Kit	Dayou	1117392
Human IL-6 ELISA Kit	Dayou	1110602
Lactate Colorimetric/Fluorometric Assay Kit	Biovision	K607-100
Acetyl-CoA Assay Kit	abcam	ab87546
CheKine™ Micro Coenzyme I NAD(H) Assay Kit	Abbkine Scientific Co., Ltd	KTB1020
CheKine™ Micro Coenzyme II NADP(H) Assay Kit	Abbkine Scientific Co., Ltd	KTB1010

JC-1 Mitochondrial Membrane Potential Assay Kit	Yeasen	40706ES60
Seahorse XF Cell Mito Stress Test Kit	Agilent	103015-100
Seahorse XF Glycolysis Stress Test Kit	Agilent	103020-100
Seahorse XF Glycolytic Rate Assay Kit	Agilent	103344-100
Hyperactive Universal CUT&Tag Assay Kit for Illumina Pro	Vazyme	TD904
Experimental Models: Cell Lines		
HUVECs	Sciencell	N/A
Experimental models: Organisms/strains		
APOE-/-（C57BL/6J-Apoeem1C/Cya）	Cyagen Biosciences Inc	S-KO-01101
Mouse: C57BL/6J	Shanghai JieSiJie Laboratory Animals Co., LTD	N/A
Software and algorithms		
GraphPad Prism 8	Graphpad Software	N/A
SPSS software (v.22.0)	SSPS Inc, Chicago, IL	N/A
TBtools	Chen C et al[1]	N/A
Bowtie2	Langmead and Salzberg[2]	N/A

IGV	Thorvaldsdottir et al[3]	N/A

Methods details

Study subjects

From July 2022 to March 2023, 110 participants with suspected OSA (snoring, witnessed apnoea or excessive daytime sleepiness, etc) were recruited randomly in the present study from the sleep center of Ruijin Hospital, Shanghai Jiao Tong University School of Medicine. All participants underwent anthropometric measurements, filling in questionnaires and subsequent overnight polysomnography (PSG).

Exclusion criteria were individuals (1) having pre-existing CVD, including coronary heart disease, heart failure, arrhythmia, or stroke etc.; (2) having the family history of CVD; (3) having the history of respiratory diseases (e.g., asthma, chronic obstructive pulmonary disease, pulmonary hypertension etc.), cerebrovascular diseases, autoimmunity diseases and malignant tumor. (4) having recent infection or concurrent use of antibiotics; (5) having received OSA treatment including continuous positive airway pressure before enrollment; and (6) inability to complete questionnaires or PSG motoring. Participants were grouped according to the apnea-hypopnea index (AHI). Non-OSA, mild to moderate OSA, and severe OSA, were defined as an AHI of <5, 5-30, and$\geq$30 events/hour, respectively. The project was approved by the ethics committee of Ruijin Hospital, which was performed in accordance with the principles of the Declaration of Helsinki. Written informed consent was obtained from all participants.

Clinical characteristics, PSG monitoring and CVD risk assessment

Anthropometric data were obtained including height, weight, body mass index (BMI), neck circumference (NC), and waist circumference (WC). Blood pressure was measured before sleep (9 PM), and in the morning (7 AM) under the supine position (Omron, HEM-7136, Japan). And hypertension was defined as a systolic blood pressure≥140 mmHg, diastolic blood pressure≥90 mmHg, or a clinical diagnosis of hypertension and current prescription of antihypertensive medication[4]. Smoking status was referred to those who regularly had smoked at least 1 cigarettes/day in the past month. Furthermore, all participants filled out the Pittsburgh Sleep Quality Index (PSQI) and Epworth Sleepiness Scale (ESS) questionnaires.

All participants underwent overnight polysomnography (Alice 6; Philips Respironics) for at least 7 hours (from 11 PM to 6 AM). Sleep recordings were scored following standard protocols recommended by the American Academy of Sleep Medicine[5]. According to the AHI, subjects were categorized into three groups, including the Non-OSA (AHI < 5), mild to moderate OSA (AHI: 5-30), and severe OSA group (AHI ≥ 30). Moreover, lowest SpO_2 ($LSpO_2$), mean SpO_2, and time spent with SpO_2 < 90% (TS90) were obtained.

CVD risk was evaluated using the Framingham 2008 algorithm, which was further categorized to low (< 5%), moderate (5-10%), and high (≥ 10%) CVD risk[6].

Biochemical measurements.

Plasma samples were collected in the morning after overnight PSG and were stored at -80°C for further analysis. Total cholesterol (TC), triglycerides (TG), high-

density lipoprotein cholesterol (HDL-C), low-density lipoprotein cholesterol (LDL-C), glucose, and uric acid (UA) were measured by colorimetric assays using a commercially available kit (Nanjing Jiancheng Bioengineering Institute). ELISA kits were used to measure concentration of PFKFB3(Aifang biological). Plasma lactate concentration was analyzed by a colorimetric L-Lactate assay kit (AAT Bioquest).

Animal models

Experiments were approved by the animal care and use committee of Ruijin Hospital, in accordance with the guidelines for Directive 2010/63/EU of the European Parliament on the protection of animals used for scientific purposes and the care and use of laboratory animals implemented by the National Institutes of Health. Male C57BL/6J mice aged 6-8 weeks (purchased from Shanghai JieSiJie Laboratory Animals Co., LTD, China) and $APOE^{-/-}$ mice on a C57BL/6 background (male, 8 weeks old, obtained from Cyagen Biosciences Inc, China) were housed under standard conditions with a 12-h light-dark cycle at 22-25°C that provided free access to water and food. C57BL/6J mice were fed with normal chow diet, and $APOE^{-/-}$ were fed with HFD (XTHF60, 61 Kcal% Fat, 21% kcal carbohydrates and 18% kcal protein, Xietong Shengwu).

Experimental Model of CIH and pharmacological intervention

The CIH exposure protocol has been described in detail previously[7]. Briefly, the mice were exposed to cages via automated, computer-controlled gas exchange

systems to achieve alternating 45s 6.5% O_2 and 45s of 21% O_2 cycles, 30 cycles h^{-1} for 8 h day^{-1} during daylight (9 AM-5PM), while normoxic control (NC) mice were exposed to air (21% O_2) in identical chambers. C57BL/6J mice were subjected to CIH for 4, 8, or 12 weeks. $APOE^{-/-}$ were exposed to NC or CIH for 8 weeks.

For pharmacological treatment, mice were administered 3PO (T3260, Targetmol) (20mg/kg; i.p. daily), SAC (T3149, Targetmol) (5mg/kg; i.p. daily), or 2-ME (HY-12033, MCE) (5 mg/kg; i.p. every two days) for 1 hour before CIH challenge.

Blood pressure, Immunofluorescent and Oil-Red-O staining

Blood pressure was consecutively measured three times in steady-state conditions using the tail-cuff method (Shanghai Meilisai Life Science Co., Ltd) and took the mean value, which was also measured every four weeks. At the end of the experimental periods, mice were anesthetized with inhaled 4% isoflurane, followed by cervical dislocation for tissue collection. Hearts and aortas were collected from the base of ascending aorta to the iliac bifurcation after removement of the adipose tissue and lymph nodes that are close to the aortas under a dissecting microscope. Hearts and 1/2 of the ascending aortas were fixed in 4% paraformaldehyde and processed for OCT embedding, from which slices of aortic root were stained with Oil Red O (Servicebio) to analyze the cross-sectional atherosclerotic lesion areas, and the quantification of atherosclerotic lesions area in aortas was performed by ImageJ.

The whole aortas were cut at the level of diaphragm into two segments: abdominal aortas and thoracic aortas. Next, thoracic aortas were cut into two equal

pieces, and the upper half parts of thoracic aortas were fixed in a final concentration of 4% paraformaldehyde and stored at 4°C for immunofluorescent or H&E staining (Servicebio). The rest of thoracic aortas were placed in liquid nitrogen and then stored at -80°C for further analysis. Abdominal aortas were prepared into single cell suspensions for flow cytometry analysis.

Immunofluorescent staining was conducted in our previous reports. Briefly, aortic sections were embedded in paraformaldehyde and cut into 10 μm slices. After washing with tris-buffered saline (TBS), the aortas were stained for PFKFB3 (Abcam, AB181661), IL-6(Servicebio, GB11117-100), ICAM-1(Servicebio, GB11106-100), PECAM-1 (R&D Systems, AF3628) and nuclei/DAPI. After washing with TBS, the sections were incubated with secondary antibodies. Next, optical samples were observed under a Leica TCS SP8 confocal microscope (Leica Microsystems, Wetzlar, Germany) and fluorescent intensity was quantified using ImageJ.

Single Cell Suspensions from Mouse Aorta and Flow Cytometry

Abdominal aortas were kept in ice-cold fluorescence activated cell sorter (FACS) buffer (DPBS+ 2% fetal bovine serum) in 6-well plates until enzyme digestion. Then, each aorta was transferred into 1ml enzyme cocktail (1mg/ml collagenase I, 1mg/ml collagenase II, 100 μg/ml DNase I, 100 μg/ml hyaluronidase in DPBS containing calcium), cut into small pieces using scissors and kept in 37 °C with slow shaking[8]. After 1 hour, the digestion process was ended by adding 1ml FACS buffer, and the digestion solution was transferred onto a 70 μm cell strainer which is placed on the

top of a new 50 ml Falcon tube. Remaining aorta tissues were mashed with syringe plunger and rinse cell strained with 3 ml FACS buffer. Cell Suspensions were centrifuged at 300g, 4 °C for 5 min. Precipitates were then resuspended in 2 ml FACS and counted under a light microscope.

Single cell suspensions were stained with a cocktail of antibodies (10^6 cells-100ul cocktail) against live-dead-FVS620, CD45 PerCp-Cy5.5, CD11b-BV510, CD11C-APC, Ly6C-BV605, Ly6G-PE-CY7, F4/80-PE, B220-BV786, CD3-PE-Cy5, CD4-FITC, CD8-APC-CY7, CD86-BV421 or BUV496 (all from BD Pharmingen). Cells were analyzed by BD LSRFortessa X-20. All flow cytometry data were analyzed using FlowJo Software (Tree Star Inc.).

Cells were identified as (1) B cells（CD45+B220+ CD3-), (2) CD4 T cells (CD45+ B220-CD3+CD4+ CD8-), (3) CD8 T cells (CD45+ B220-CD3+CD4-CD8+), (4) granulocytes (CD45+CD11C-CD11b+ Ly6G+), (5) monocytes (CD45+CD11C-CD11b+ Ly6G-F4/80–), (6) macrophages (CD45+CD11C-CD11b+ Ly6G-F4/80+), (7)Ly6Chi monocytes (CD45+C11C-CD11b+ Ly6G-Ly6Chigh), (8) Ly6Cint monocytes (CD45+CD11C-CD11b+ Ly6G-Ly6Cinter), and (9) Ly6Clow monocytes (CD45+CD11C-CD11b+ Ly6G-Ly6Clow).

As for dimensionality reduction, analysis, t-distributed stochastic neighbor embedding (t-SNE) was analyzed on live, CD45+ leukocytes based on canonical phenotyping markers. A subset of 900 cells was selected from each sample by using Downsample algorithm of FlowJo at random and concatenated into a single population prior to t-SNE analysis. T-SNE analysis was performed using 3000

iterations, a perplexity of 100, a learning rate of 200, and a theta of 0.5. T-SNE dimension X and dimension Y were generated by plotting each event by its t-SNE dimensions in a dot-plot. Cell populations defined by the manual gating strategy were projected onto t-SNE maps and assigned specific colors.

Administration of AAV

For in vivo vascular endothelial-specific PFKFB3 overexpression or knockdown, custom-made adeno-associated viral vector (HBAAV2/VEC) carrying mouse Pfkfb3 (79050235) or mir30 targeting mouse Pfkfb3 (79050237) with a Tie promoter and its negative control (AAV-control) were obtained from Hanbio (Hanbio, Inc., Shanghai, China)[9]. A single injection of AAV-Pfkfb3(High), AAV-Pfkfb3(Low), or AAV-control at a dose of $1*10^{11}$ viral genomes was administrated into mice via intravenous injection. Bioluminescence imaging was performed at 4 weeks after AAV injection, following the instructions of the IVIS Lumina XR small animal optical imaging system (PerkinElmer). Primary aortic ECs and non-ECs were isolated using CD31 magnetic beads (Miltenyi Biotec), and Pfkfb3 expression was assessed by Western blot.

Cell culture

Primary human umbilical vein endothelial cells (HUVECs) were purchased from ScienCell and grown in endothelial cell medium (ECM) supplemented with 5% FBS, 1% Penicillin/Streptomycin and the supplement pack (ScienCell, 1001) in a

humidified atmosphere of 21% O_2 and 5% CO_2 at 37°C. Cells between passage 3-7 were used for experiments. THP-1 cells (obtained from the Shanghai Institute of Immunology) were cultured in RPMI-1640 supplemented with 10% FBS and 1% Penicillin/Streptomycin.

For transductions, HUVECs were infected with either pLV[Exp]-EGFP:T2A:Puro-EF1A>FLAG/hPFKFB3[NM_001363545.2] (pLV-PFKFB3) for PFKFB3 overexpression, or pLV[shRNA]-EGFP:T2A:Puro-U6>hPFKFB3 (pLV-shPFKFB3) to silence PFKFB3 expression, or pLV-control followed by antibiotic selection. Lentivirus was generated by VectorBuilder. A multiplicity of infection (MOI) of 10 was used in all experiments. Cells were transduced overnight in the presence of 0.5 μg/ml polybrene and re-fed with fresh medium the next day. After antibiotic selection, the efficacy of over expression or knockdown was verified by Real Time quantitative polymerase chain reaction (RT-qPCR) analysis and fluorescence microscope observations.

For RNAi experiments, HUVECs were transfected with HIF-1α siRNA or negative control siRNA (Aisen) using Lipofectamine 3000 (Invitrogen) according to the manufacturer's instructions.

CIH exposure and plasma stimulation model

The model of CIH in HUVECs was described previously[7]. Briefly, HUVECs were exposed to cages where O_2 concentration oscillated between 1% O2 for 5 min and 21% O2 for 5 min via the automated, computer-controlled gas exchange system

(BioSpherix-OxyCycler-42, Redfield, NY) for 24h.

For the plasma stimulation model, HUVECs were incubated with 20% plasma (with ECM) from Non-OSA (n=10), mild to moderate OSA (n=10), and severe OSA (n=10), which were age, sex and body mass index matched. Cells were harvested after 6h for RT-qPCR analysis. After exposing to plasma medium for 24 h, cells were collected for Seahorse Flux analysis.

In experiments including inhibitors, the inhibitors 2ug/mL 3PO (MCE, HY-19824), 10uM SAC (MCE, HY-N0319), 1uM 2-ME (MCE, HY-12033), 10 mM Oxamate (MCE, HY-W013032A), or 10 mM 2-deoxyglucose (2DG, Agilent) were added to the ECM intended for treatment 1 h prior to CIH or plasma incubation experiments.

Total RNA isolation, RNA sequencing and RT-qPCR

Total RNA was extracted using RNAiso (TAKARA, 9109) and further treated with DNase to remove genomic DNA contamination following the manufacturer's instructions. The quality of RNA was analyzed, and then, cDNA libraries were then constructed. In this project, 6 samples were sequenced used DNBSEQ platform, averagely generating about 1.19G Gb bases per sample (BGI, F21FTSECWGT0094_HUMfxptN]). Reads were then filtered and aligned to the human genome version hg38 (GCF_000001405.39_GRCh38.p13). Differentially expressed genes (DEGs) with a false discovery corrected p <0.05 were used for

further analysis (heatmaps and functional enrichment analysis) on the platform of Dr.Tom (BGI). Heatmaps were made by TBtools.

For RT-qPCR, 1 μg of total RNA was used for cDNA synthesis (Yeasen, 11141ES60). RT-qPCR was performed using SYBR Premix Ex Taq (Yeasen, 11202ES08) on a ViiA7 PCR machine. Primer sequences are outlined in Supplementary Table3. Gene expression was normalized to reference gene β-actin, and graphs indicated fold change of relative gene expression of which values were normalized to the mean of the NC group.

Western blot analysis

Proteins prepared from mouse aortas or cell lysates were collected for Western blot analysis. Proteins were electrophoresed through sodium dodecyl sulfate polyacrylamide gels, and transferred to polyvinylidene difluoride membranes (Cytiva, 10600023). Blots were incubated with primary antibodies and signals were visualized using the Immobilon Western HRP Substrate (Millipore). The bands were quantified by Image J, and expression levels were normalized against β-actin. Primary antibodies included anti-β-actin (1:1000, Servicebio), anti-IL-6 (1:1000, Aifang biological) , anti-E-selectin (1:1000, Beyotime), anti-ICAM-1 (1:1000, Beyotime), anti-EDN1 (1:500, Proteintech), anti-HK2 (1:1000, Santa Cruz), anti-LDHA (1:1000, Santa Cruz), anti-PFKFB3 (1:1000, abcam), and anti-HIF-1α (1:500, Santa Cruz).

Monocyte-endothelial adhesion assay

48well plates were included 2×10^4 HUVECs per well. Having reached about 70-80% confluency, the medium was changed for fresh ECM and exposed to NC or CIH in the presence or absence of inhibitors. After 24h of incubation, the medium was renewed. 4×10^5 THP-1 cells per well were added into plates incubated at 37°C in 5% CO_2 for 1 h, followed by washing to remove non-adhered monocytes. The number of adhered monocytes was counted using Zeiss Axio Vert.A1 microscope (Zeiss, Germany)

Cytokines and metabolic measurements

Cytokine production was measured in supernatants of HUVECs using the cytometric bead array by flow cytometry (RayBiotech), or ELISA kits for IL-6, CXCL8, CCL2, and ICAM-1(see Supplementary Materials). HUVECs exposed to NC or CIH in the presence or absence of inhibitors were extracted for metabolites measurements, including lactate (Biovision, K607-100), Acetyl-CoA (abcam, ab87546), NAD^+/NADH (Abbkine Scientific Co., Ltd, KTB1020), NADPH and $NADP^+$/NADPH (Abbkine Scientific Co., Ltd, KTB1010) according to the manufacturer's protocol.

2-NBDG uptake assays, ROS and flow cytometry

After 24h of NC or CIH incubation, ECM was replaced with fresh ECM without FBS. 2-NBDG (Thermofisher, N13195) was added in a final concentration of 20ug/ml and HUVECs were incubated at 37°C in 5% CO_2 for 1 h, followed by

washing twice with pre-cooled PBS. Cells were digested and resuspended in 200ul FACS for flow cytometry. As for ROS measurements, cells were incubated in PBS contained 5μM CELLROX GREEN REAGENT (ThermoFisher, C10444) at 37℃ for 30min. Cells were then washed by pre-cooled PBS for 3 times and remained on ice prior flow cytometric measurements. Fluorescence was measured in the FITC channel on an LSR-Fortessa X20 flow cytometer (BD, USA).

Seahorse Flux Analysis

The Seahorse XFe96 analyzer (Agilent Technologies) was used for bioenergetic analysis. After NC or CIH in the presence or absence of inhibitors, HUVECs were seeded in the Seahorse XF96 microplates (Agilent, 102601-100) at 3×10^4 cells/well/80 μL and plated 1 day prior to assay. On the next day, cells were washed by Glycolysis Stress Test assay medium（Seahorse XF DMEM Medium (Agilent, 103575-100) supplement with 2 mM glutamine (Agilent, 103579-100)) or Mito Stress Test assay medium（Seahorse XF DMEM Medium supplement with 2 mM glutamine,10 mM glucose (Agilent, 103577-100), 1 mM sodium pyruvate, (Agilent, 103578-100)), and incubated for 1 h in a non-CO_2 incubator at 37 °C before a final wash in the assay media.

Extracellular acidification rates (ECAR) were measured by Seahorse XF Glycolysis Stress Test Kit (Agilent, 103020-100), followed by sequential injection of glucose (10mM), oligomycin (1μM), and 2-Deoxy-D-glucose (2-DG; 50mM). The indexes of

glycolytic function were obtained, including Glycolysis, Glycolytic capacity, Glycolytic reserve and Non-glycolytic acidification

OXPHOS was determined by Seahorse XF Cell Mito Stress Test Kit (Agilent, 103015-100). OCR changes were measured in response to oligomycin (1 μM), FCCP (1μM) and 0.5 μM rotenone + 0.5 μM antimycin A injection. The indexes of mitochondrial function were obtained, including basal respiration, ATP-linked respiration, maximal respiration, non-mitochondrial oxygen consumption, etc.

For the plasma stimulation model, 3× 104 cells were seeded in the Seahorse XF96 microplates. After 24 h, HUVECs were incubated in ECM with 20% plasma from different group. After 24 h, cells were washed in assay media and analyzed using Seahorse XF Glycolytic Rate Assay Kit (Agilent, 103344-100). The Seahorse XFe96 analyzer was operated using the WAVE Software (V2.6.1, Agilent Technologies) and assay standard drug injections were used of 0.5 μM rotenone + 0.5 μM antimycin Ain port A and 50mM 2-DG in port B.

CUT&Tag-Seq and CUT&Tag-qPCR

CUT&Tag was performed by Hyperactive Universal CUT&Tag Assay Kit for Illumina Pro (TD904, Vazyme) according to the manufacturer’s instructions. Briefly, HUVECs were collected and bound to ConA beads Pro. Then cells were resuspended in antibody buffer and incubated with primary antibodies against H3K18la or IgG, and secondary antibodies in order. Subsequently, the samples were incubated with pA/G-Tnp transposase. After tagmentation, DNA was extracted, amplified, and

purified to construct library (N411, Vazyme). The library was quantified and sequenced on an Illumina HiSeq/Novaseq instrument (Illumina, San Diego, CA, USA). For analysis of the CUT&Tag data, clean reads were aligned to the human reference genome (GRCh38.109) via software Bowtie2 (version 2.2.6). Peaks quality control, peaks calling and peaks annotation were analyzed by MACS (V2). For data visualization, the Integrative Genomics Viewer (IGV) was utilized.

For CUT&Tag-qPCR, DNA was extracted and incubated with Stop Buffer at 95℃ for 5 min. qPCR was performed using SYBR Premix Ex Taq (Yeasen, 11202ES08) on a ViiA7 PCR machine. The primer sequences for CUT&Tag-qPCR are listed in Supplementary Table.2.

Luciferase reporter assay

To assess the functional role of H3K18la in PFKFB3 transcription, a dual-luciferase reporter assay was performed. The wild-type (WT) PFKFB3 promoter sequence and a mutant (MUT) version lacking putative H3K18la-responsive elements were cloned into the pGL4-Basic luciferase reporter vector (Promega). HUVECs were co-transfected with WT or MUT reporter constructs and a Renilla luciferase control vector (pRL-TK, Promega) using Lipofectamine 3000 (Invitrogen). After 48 h, cells were exposed to NC or CIH for an additional 24 h, with or without oxamate or 2DG treatment. Luciferase activity was measured using the Dual-Luciferase Reporter Assay System (Invitrogen) and normalized to Renilla activity.

Bioinformatics analysis

Correlation analysis of gene expression of aortas was based on the Genotype-Tissue Expression (GTEx) database using the online tool GEPIA (Gene Expression Profiling Interactive Analysis) (http://gepia.cancer-pku.cn/).

Single-cell RNA sequencing (scRNA-seq) analysis was based on the GSE159677 dataset, which obtained atherosclerotic core plaques and patient-matched proximal adjacent portions of carotid artery tissue from three patients undergoing carotid endarterectomy[7]. Raw expression matrices of arterial scRNA-seq data were downloaded under the accession number, GSE159677[10]. The data was analyzed by the R package 'Seurat' (v4.0.2)[11], which was used to perform filtering, normalization, dimensionality reduction, clustering, and differential expression analysis. Cell clusters were annotated by using both the R package 'SingleR' (v1.6.1)[12]. Next, ECs with above-average PFKFB3 expression were defined as PFKFB3-high ECs, and the rest as PFKFB3-low ECs. Differentially expressed genes were identified with adjusted p values less than 0.05.

Statistical analysis

Statistical analyses were performed using GraphPad Prism (v.8.0, GraphPad Software, La Jolla, CA, USA) and SPSS software (v.22.0, SSPS Inc, Chicago, IL). Normal distribution was assessed using the Kolmogorov–Smirnov test. Continuous variables with a normal distribution were presented as means ± standard error of the mean (SEM), while values without a normal distribution are presented as median (25%-75%). According to the distribution, Student's t tests, Paired t-test, one-way analysis

of variance (ANOVA) or the Kruskal–Wallis test was used for comparisons among groups. Categorical variables are presented as numbers and percentages, and were analyzed using Fisher's exact test. Pearson's correlation analysis or Spearman rank correlation was used investigate the correlation. Statistical significance was reported as follows: * $P < 0.05$, ** $P < 0.01$, *** $P < 0.001$.

References:

1. Chen C, Wu Y, Li J, Wang X, Zeng Z, Xu J, et al. TBtools-II: A “one for all, all for one” bioinformatics platform for biological big-data mining. Molecular Plant. 2023; 16: 1733-42.

2. Langmead B, Salzberg SL. Fast gapped-read alignment with Bowtie 2. Nature Methods. 2012; 9: 357-9.

3. Thorvaldsdottir H, Robinson JT, Mesirov JP. Integrative Genomics Viewer (IGV): high-performance genomics data visualization and exploration. Briefings in Bioinformatics. 2012; 14: 178-92.

4. Whelton PK, Carey RM, Aronow WS, Casey DE, Jr., Collins KJ, Dennison Himmelfarb C, et al. 2017 ACC/AHA/AAPA/ABC/ACPM/AGS/APhA/ASH/ASPC/NMA/PCNA Guideline for the Prevention, Detection, Evaluation, and Management of High Blood Pressure in Adults: Executive Summary: A Report of the American College of Cardiology/American Heart Association Task Force on Clinical Practice Guidelines. Circulation. 2018; 138: e426-e83.

5. Berry RB, Brooks R, Gamaldo C, Harding SM, Lloyd RM, Quan SF, et al. AASM Scoring Manual Updates for 2017 (Version 2.4). J Clin Sleep Med. 2017; 13: 665-6.

6. D'Agostino RB, Sr., Vasan RS, Pencina MJ, Wolf PA, Cobain M, Massaro JM, et al. General cardiovascular risk profile for use in primary care: the Framingham Heart Study. Circulation. 2008; 117: 743-53.

7. Yan YR, Zhang L, Lin YN, Sun XW, Ding YJ, Li N, et al. Chronic intermittent hypoxia-induced mitochondrial dysfunction mediates endothelial injury via the TXNIP/NLRP3/IL-1beta signaling pathway. Free Radic Biol Med. 2021; 165: 401-10.

8. Hu D, Yin C, Mohanta SK, Weber C, Habenicht AJ. Preparation of Single Cell Suspensions from

Mouse Aorta. Bio Protoc. 2016; 6.

9. Zeng H, Pan T, Zhan M, Hailiwu R, Liu B, Yang H, et al. Suppression of PFKFB3-driven glycolysis restrains endothelial-to-mesenchymal transition and fibrotic response. Signal Transduct Target Ther. 2022; 7: 303.

10. Alsaigh T, Evans D, Frankel D, Torkamani A. Decoding the transcriptome of calcified atherosclerotic plaque at single-cell resolution. Commun Biol. 2022; 5.

11. Cao YX, Fu LY, Wu J, Peng QK, Nie Q, Zhang J, et al. Integrated analysis of multimodal single-cell data with structural similarity. Nucleic Acids Research. 2022; 50.

12. Long ZL, Sun CF, Tang M, Wang Y, Ma JY, Yu JC, et al. Single-cell multiomics analysis reveals regulatory programs in clear cell renal cell carcinoma. Cell Discov. 2022; 8.
